# Target Capture Reveals the Complex Origin of Vietnamese Ginseng

**DOI:** 10.3389/fpls.2022.814178

**Published:** 2022-07-13

**Authors:** Hien Thi Thu Le, Linh Nhat Nguyen, Hang Le Bich Pham, Hao Thi My Le, Toan Duc Luong, Hue Thi Thu Huynh, Van Tuong Nguyen, Hai Van Nong, Irene Teixidor-Toneu, Hugo J. De Boer, Vincent Manzanilla

**Affiliations:** ^1^Institute of Genome Research, Vietnam Academy of Science and Technology, Hanoi, Vietnam; ^2^Graduate University of Science and Technology, Vietnam Academy Science and Technology, Hanoi, Vietnam; ^3^Soils and Fertilizers Research Institute, Vietnam Academy of Agricultural Sciences, Hanoi, Vietnam; ^4^Institute of Biotechnology, Vietnam Academy of Science and Technology, Hanoi, Vietnam; ^5^Natural History Museum, University of Oslo, Oslo, Norway; ^6^Baseclear BV, Leiden, Netherlands

**Keywords:** crop domestication, *Panax*, population genomics, Vietnam, target capture sequencing

## Abstract

The global market of the medicinal plant ginseng is worth billions of dollars. Many ginseng species are threatened in the wild and effective sustainable development initiatives are necessary to preserve biodiversity at species and genetic level whilst meeting the demand for medicinal produce. This is also the case of *Panax vietnamensis* Ha & Grushv., an endemic and threatened ginseng species in Vietnam that is locally cultivated at different scales and has been the object of national breeding programs. To investigate the genetic diversity within cultivated and wild populations of *P. vietnamensis* we captured 353 nuclear markers using the Angiosperm-353 probe set. Genetic diversity and population structure were evaluated for 319 individuals of Vietnamese ginseng across its area of distribution and from wild and a varying range of cultivated areas. In total, 319 individuals were sampled. After filtering, 1,181 SNPs were recovered. From the population statistics, we observe high genetic diversity and high genetic flow between populations. This is also supported by the STRUCTURE analysis. The intense gene flow between populations and very low genetic differentiation is observed regardless of the populations' wild or cultivated status. High levels of admixture from two ancestral populations exist in both wild and cultivated samples. The high gene flow between populations can be attributed to ancient and on-going practices of cultivation, which exist in a continuum from understorey, untended breeding to irrigated farm cultivation and to trade and exchange activities. These results highlight the importance of partnering with indigenous peoples and local communities and taking their knowledge into account for biodiversity conservation and sustainable development of plants of high cultural value.

## Introduction

Ginseng has been used in traditional medicine in China for thousands of years (Robbins, 1998). Today, ginseng is used collectively to refer to several plant species, mainly in the Araliaceae genera *Panax* L. and *Eleutherococcus* Maxim. The economic value of ginseng in the global medicinal plant trade is estimated to be in excess of US$2.1 billion (Baeg and So, 2013). The most commonly used species in the genus are *Panax ginseng* Meyer (Korean ginseng), *P. quinquefolius* L. (American ginseng), *P. notoginseng* (Burkill) F. H. Chen (Chinese ginseng), *P. japonicus* (T. Nees) C. A. Mey. (Japanese ginseng), *P. pseudoginseng* Wall. (Himalayan ginseng) and *P. vietnamensis* Ha and Grushv. (Vietnamese ginseng) (Yang, 2021). The majority of commercialized ginseng material is from cultivation and controlled sustainable wild harvest, whereas material from uncontrolled depletive wild harvesting seems to play a minor and decreasing role (Ichim and de Boer, 2020). So far, academic enquiry has unproportionally focused on *P. ginseng, P. quinquefolius*, and *P. notoginseng* (Zhou et al., 2005; Pan et al., 2016; Xia et al., 2016; Fan et al., 2020). Less is known of the genetic and chemical variation of other ginseng species and whether cultivation predominates over wild harvesting (Zhou et al., 2020).

In Vietnam, a number of ginseng species occur in the wild. The most recent studies recognize five taxa including three species, *P. bipinnatifidus* Seem., *P. stipuleanatus* H. T. Tsai and K. M. Feng and *P. vietnamensis* Ha and Grushv. and two varieties, *Panax vietnamensis* var. *fuscidiscus* K. Komatsu, S. Zhu and S. Q. Cai (Zhu et al., 2003; Nguyen, 2005; Phan et al., 2013) and *P. vietnamensis* var. *langbianensis* Duy, V. T. Tran and L. N. Trieu (Nong et al., 2016). *P. pseudoginseng* Wall (Lào Cai, Hà Giang, and Cao B
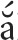
ng provinces) and *P. ginseng* Meyer (Lào Cai province) are species that were imported for cultivation, but are rarely found in the wild (Nguyen, 2005). The three wild species are referenced in the Vietnam Red Data Book as threatened (Dang, 2006). The locally occurring *P. vietnamensis* has a long history of use, but it was only described as a species new to science in 1985 (Ha and Grushvitzky, 1985). In Vietnamese, this species has the vernacular names “sâm Ngọc Linh” (“sâm” = ginseng), “sâm Việt Nam,” “sâm khu Năm,” “sâm đốt trúc,” “c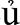 Ngải rọm,” or “Thuốc giấu”. The complete area of distribution of *P. vietnamensis* is unknown, but in Vietnam it is found wild in the Ngọc Linh mountains straddling the provinces of Quảng Nam and Kon Tum. Vietnamese ginseng had long been used by the local X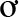 Ðăng ethnic group living at the foot of Mount Ngọc Linh, who used it to treat a variety of illnesses (Dang, 1999). For the last few decades, a growing interest in development of genetic resources and self-sufficiency drove domestic bioprospecting. In 1973, pharmacists Dao Kim Long and Nguyen Chau Giang discovered a *Panax* species locally known as ‘sâm đốt trúc' growing at a height of 1,800 m in the Kon Tum province, and published it as *Panax articulates* K. L. Dao *nom. inval*. Ha Thi Dung and Grushvitzky later formally described the species as *P. vietnamensis* Ha and Grushv. based on samples collected from this area (Ha and Grushvitzky, 1985). This new ginseng was promoted as a resource to treat wounded soldiers, miners, and local people. In 1974, a preliminary comparative analysis of the chemical composition of this Vietnamese ginseng with Asian and American ginseng showed that it was rich in ginsenosides (Dao and Nguyen, 1991). Subsequent pharmacological studies corroborated its health benefits and resulted in a wide awareness of the species through mass media in Vietnam. The exploitation, commercialization and use of this Vietnamese ginseng boomed in the 1980s and led to a sharp decrease in availability of material sourced from the wild (Dao and Nguyen, 1991). From 1979, cultivation studies have been conducted in a more systematic manner at Trà Linh Medicinal Plant Center (Quảng Nam province) by propagating plants both asexually and sexually, increasing its cultivation area, and through encouraging ethnic minorities to plant cultivars (Dao and Nguyen, 1991).

Several studies have investigated the relations of *P. vietnamensis* to other ginseng species. In 2001, Komatsu et al. compared the genetic characteristics of *P. vietnamensis* based on 18S and the *matK* gene sequences and showed that it was completely homogeneous between *P. vietnamensis* and *P. quinquefolius* on the 18S gene, but there was a difference of 10 nucleotides on the *matK* gene (Komatsu et al., 2001). In 2003, Zhu et al. described a new sub-species of *P. vietnamensis* distributed in Yunnan, China, and named it *P. vietnamensis* var. *fuscidiscus* K. Komatsu, S. Zhu and S. Q. Cai. This new subspecies is different from *P. vietnamensis* at 4 nucleotides located on the *trnK* gene (1 nucleotide on the 5′ extended region, 2 nucleotides on the *matK* gene and 1 nucleotide on the 3′ extended region). In 2016, Nong et al. described a new variety *P. vietnamensis* var. *langbianensis* Duy, V. T. Tran and L. N. Trieu from the Lam Vien plateau, and a later ISSR study of 115 individuals from two populations showed it to have high genetic diversity (Le et al., 2019). Recent molecular studies using whole plastome data have shown that *P. vietnamensis* is sister to the widespread *P. japonicus* which ranges from India to Japan (Manzanilla et al., 2018). Other studies have investigated the differentiation between *P. vietnamensis* and *P. ginseng* (Vasyutkina et al., 2018). Studies on *P. vietnamensis* continue to shed new light on intra- and interspecific evolutionary relationships (Nguyen et al., 2020; Vu et al., 2020).

The short documented cultivation history of *P. vietnamensis* shows a patchwork of wild populations, nurseries, woodlot cultivation, and cultivation farms. Given this complex socio-ecological context, this study investigates the population genomics of wild and cultivated *P. vietnamensis*. Specifically, we enquire if wild and cultivated populations are distinct genetically. We part with two contrasting hypotheses: (1) a loss in genetic diversity in cultivars is due domestication processes, or (2) wild and cultivated plants cannot be distinguished genetically due to local plant population management practices whereby local farmers supplement their cultivated stock with wild specimens or favor reproduction between wild and cultivated specimens through woodlot cultivation. Our study has implications for biodiversity conservation best practices that involve local farmers as actors for *P. vietnamensis in-situ* and *ex-situ* conservation.

## Materials and Methods

### Study Sites

**Figure 1:**
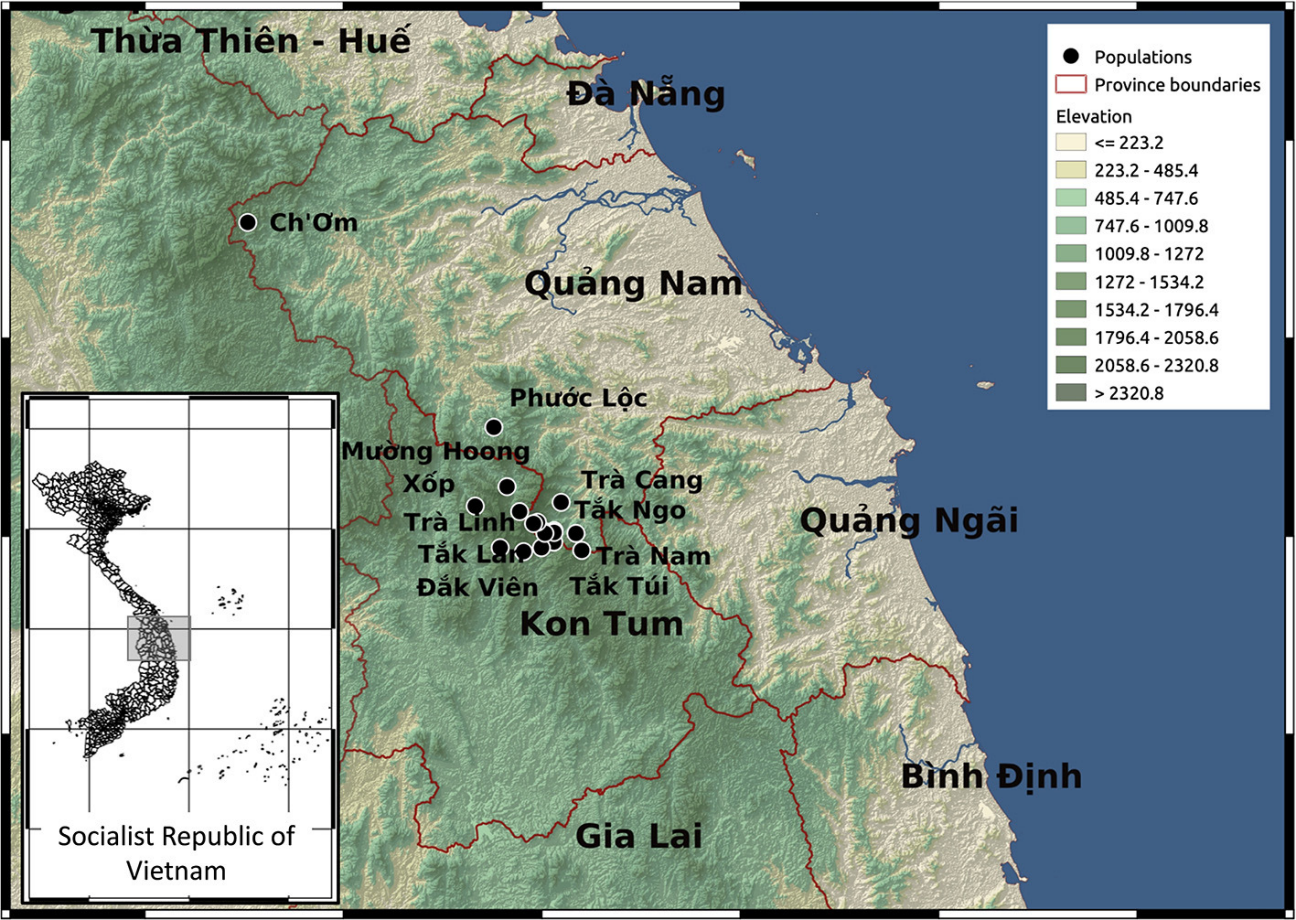
Topographic map showing the provinces and study sites in Vietnam.

Previous studies identified *P. vietnamensis* as a narrow endemic occurring only on Ngọc Linh mountain under the canopy of natural forests (Nguyen, 2005; Dang, 2006). It is the ginseng species with the southernmost distribution and has different chemical constituents compared to other *Panax* species (Yamasaki, 2000; Le et al., 2015). The Ngọc Linh mountain area covers 18 communities in five districts including Muòng Hoong, Ngọc Linh, Xốp (Ð
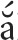
k Glei district, Kon Tum province), Ð
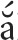
k Na, Mǎng Ri, Ngọc Lây, Ngọc Yêu, Tê Xǎng, Văn Xuôi (Tu M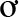 Rông district, Kon Tum province) and Trà Cang, Trà Don, Trà Don, Trà Leng, Trà Linh, Trà Nam, Trà Tập (Nam Trà My district, Quảng Nam province), Ch'om (Tây Giang district, Quảng Nam province), Ph
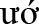
c Lộc (Ph
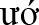
c S
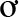
n district, Quảng Nam province). In this study, collected sample sites include: M
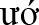
ng Hoong, Ngọc Linh and Xốp commutes (Ðǎk Glei district, Kon Tum province); Trà Cang, Trà Linh, Trà Nam commutes (Nam Trà My district, Quảng Nam province); Ch'om (Tây Giang district, Quảng Nam province); Ph
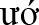
c Lộc (Ph
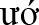
c S
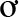
n district, Quảng Nam province) (Figure 1; Supplementary Tables S1, S2).

### Plant Sampling

*P. vietnamensis* has an erect posture with green or slightly purple colored stems. The tuberous root has a spindle shape with 2.4–4 cm long and diameter of 1.5–2 cm. The outer surface of the roots has a brown or yellowish gray color. The root has a solid body which is hard to break. Leaf and/or root samples of 319 individuals of *P. vietnamensis* were collected in the Ngọc Linh mountains in Quảng Nam and Kon Tum provinces of which 293 came from cultivars (both from hamlets and the Trà Linh Medicinal Plant Center, representing 265 leaf and 28 root samples) and 27 were wild (19 leaf and 8 root samples; Supplementary Tables S1, S2). These samples belonged to 19 populations, including five samples harvested from the wild and 14 from cultivated populations, including the Trà Linh Medicinal Plant Center and the Ngọc Linh Ginseng Center of Nam Trà My district (Supplementary Tables S1, S2). Leaf samples were preserved in silica-gel within 24 h after collecting.

### Library Preparation, Target Enrichment, and Sequencing

Genomic DNA from 319 *P. vietnamensis* samples was extracted using the GeneJET Plant Genomic DNA Purification Kit (ThermoFisher Scientific, USA) following the manufacturer's standard protocol for lignified, polyphenol-rich plant tissues. Extracted total DNA was quantified for integrity and quality by using gel electrophoresis and a NanoDrop 2000 spectrophotometer (ThermoFisher Scientific, USA). Colored and impure samples were cleaned with NEB Monarch Genomic DNA Purification Kit (New England Biolabs, USA). We assessed the DNA integrity on a Fragment Analyzer (Advanced Analytical Technologies, USA) with DNF-488 High Sensitivity Genomic DNA Analysis Kit (Supplementary Table S1). Dual indexed libraries were prepared using the protocol of Meyer and Kircher (2010). We targeted 353 low copy nuclear genes using the Angiosperm 353 probe set described in Johnson et al. (2018). An advantage of this approach is that the reduced representation library approach of target capture reduces the total sequencing costs of the project, while at the same time generating re-usable data for other studies employing this kit. Moreover, this kit has been used for population level study (Van Andel et al., 2019; Wenzell et al., 2021). We prepared and pooled 319 equimolar libraries in 21 capture reactions with an average 250 ng of input DNA per pool. The RNA probes were hybridized for 24 h before target baiting, and 16 PCR cycles were carried out after enrichment following the MyBaits v.3 manual. The enriched libraries were pooled in equimolar amounts and sequenced on two Illumina HiSeq 3000 lanes (150 bp paired-end).

### Bioinformatics Analysis

Raw sequencing reads were checked for quality using FastQC v0.11.9 (Andrews, 2010) and MultiQC v1.9 (Ewels et al., 2016). Trimmomatic v0.36 (Bolger et al., 2014) was used to remove adapter sequences and to filter low quality bases with a sliding window of 10 and mean quality equals 20 and a minimum read length of 20 bp. Trimmed reads were mapped by using the BWA_MEM algorithm in BWA v0.7.17 (Li and Durbin, 2010). The 353 supercontigs from *Aralia cordata* were used as reference (Johnson et al., 2018). Coverage of each marker for each sample was determined by BEDtools v2.29.2 (Quinlan, 2014). Following the recommendations from Manzanilla et al. (2021), markers with coverage lower than 100X were excluded from the dataset. Duplicate reads were marked and removed from BAM files with the MarkDuplicates program in Picard v2.23.1 (Wysoker et al., 2015). Single nucleotide polymorphism (SNP) calling was performed using DeepVariant v1.1.0 (Ip et al., 2020). We performed additional filtering steps using vcftools v0.1.16 (Danecek et al., 2011) and retained SNPs with mapping quality above 50, with mean depth values ranging from 30 to 500, with minor allele frequency ≥0.10, and with missing data <20%. The filtering resulted in a final dataset of 317 markers with total length equal to 570,100 bp for 282 samples. For reproducibility purposes, all the scripts used during the data processing are available on Open Science Framework (https://osf.io/w9mgc/) and on GitHub (https://github.com/vincentmanz/Ginseng), all the sequencing data have been deposited under the NCBI BioProject accession (BioProject PRJNA788747).

### Population Structure

Population structure was inferred based on 1,181 SNPs from the 317 target capture markers resulting from the filtering described above using STRUCTURE v2.3.4 (Pritchard et al., 2000). Prior analyses, VCF files format was converted with PGDSpider v2.1.1.5 (Lischer and Excoffier, 2012). STRUCTURE analyses were done using the correlated allele frequency method by defining prior population structure or location. Population structure was inferred by estimating the optimum number of clusters (*K*) values ranging from one to 20.

Many of the 317 markers have multiple SNPs and extracting multiple SNPs per marker yields blocks of closely linked SNPs. The STRUCTURE program permits inclusion of weakly linked SNPs with some degree of non-independence. To overcome the possible effects of linked SNPs, we sub-sampled our data set. From the original data set, we created 10 sets where we randomly choose 1 SNP per marker. To analyze these 10 data sets, we used the same pipeline as for the original data set, using STRUCTURE with 3 replicates and for *k* = 1 to *k* = 8 for 1,000,000 generations. Then we compiled results for the 10 data sets to estimate the best number of ancestral populations. We performed a paired *t*-test to evaluate if the populations' admixture values from the randomization set were significantly different from those obtained for the full SNPs data set. Runs were set with 1,000,000 iterations and a burn-in of 100,000. Selection of the *K* value based on the calculation of delta *K* was performed with STRUCTURE HARVESTER v0.6.94 (Earl and VonHoldt, 2012; Supplementary Figure S1). CLUMPP v1.1.2 (Jakobsson and Rosenberg, 2007) was used to obtain the average permuted individual and population Q-matrices throughout the three replicates for each *K*-value. Those matrices were used as input for distruct v1.1 (Rosenberg, 2004) which was used to obtain bar plots where each individual is represented as a segment divided into *K* colors that represent the estimated membership coefficients from each cluster.

Principal component analysis (PCA) of the multilocus genotypes was conducted to visualize potential groupings of the 282 individuals using R package “genesis” (Gogarten et al., 2019). Population statistics were obtained using the R package “hierfstat” (Goudet, 2005). Based on the filtered SNPs, we computed basic statistics, the observed heterozygosity (H_o_) and mean diversity (H_S_) within populations. Among populations, we estimated the total genetic diversity (D_ST_), and the corrected D_ST_ (D_STP_). F_ST_ and corrected F_ST_ (F_STP_) were assessed as well as F_IS_ following Nei per overall loci. We measured the population differentiation as defined by Jost (D_EST_). Finally, we estimated the overall gene diversity (H_T_) and the corrected H_T_ (H_TP_). We visualized metadata related to the population geographic situation (populations, comune, district, province) and to the cultivation status (wild or cultivated) on the PCAs, and assessed the correlation between the metadata and the principal components using the R package *PCAtools*.

## Results

### Sequencing and SNP Calling

Sequencing of the 353 targeted regions in 319 *P. vietnamensis* samples yielded a total of 1.359 billion reads from the two sequencing lanes combined for all the samples. After trimming, on average, 3.8 millions of reads per sample were retained (Supplementary Material) with a duplication level of 83%. Thirty-seven individuals and 36 nuclear markers presented a coverage under the threshold and were discarded (Supplementary Figure S2). The final dataset consists of 317 markers with total length equal to 570,100 bp for 282 samples. The SNPs pipeline analysis provided 1,181 SNPs with 8.39% of missing data on average.

### Genetic Diversity

**Figure 2:**
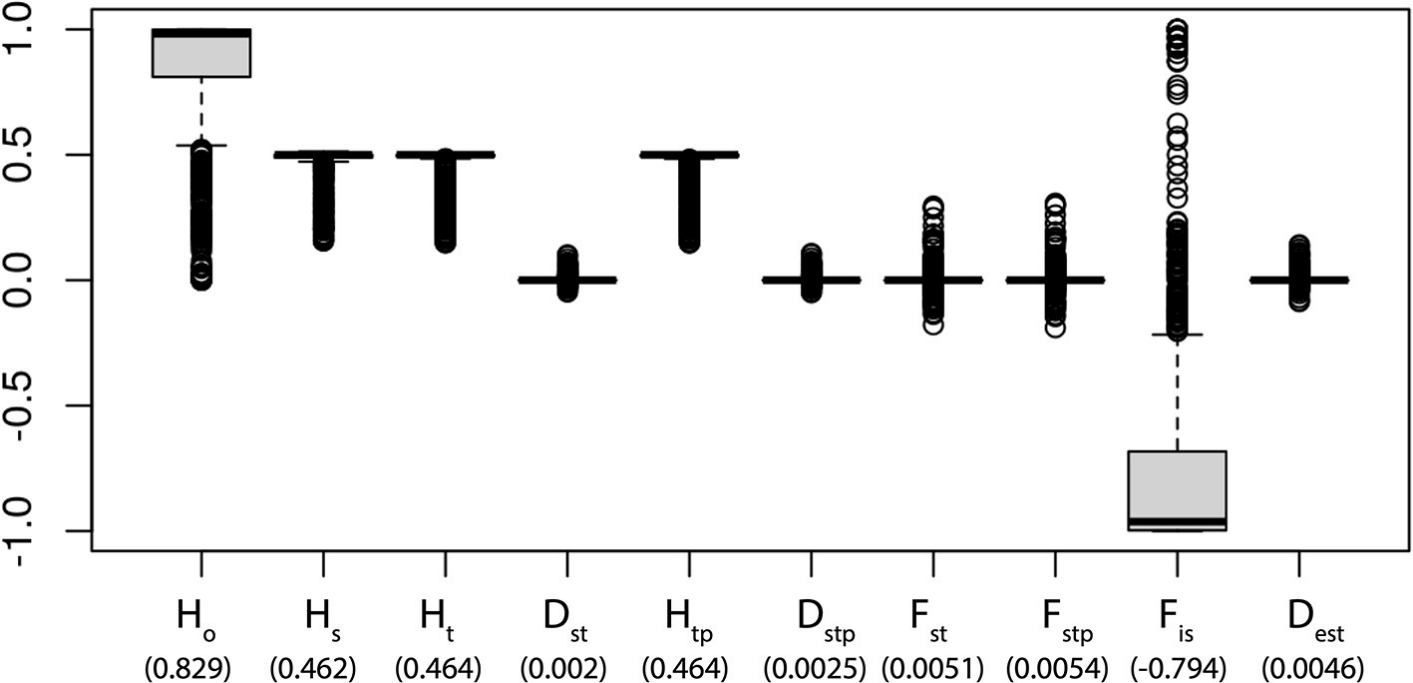
Summary statistics of genetic variation existing in *Panax vietnamensis* by 1,181 SNPs. The vertical line = median; boxes show quantiles; and points show outliers. H_O_, heterozygosity within population; H_S_, genetic diversity within population; H_T_, overall gene diversity; H_TP_, corrected H_T_; D_ST_, gene diversity among samples; D_STP_, corrected D_ST_; F_ST_, fixation index; F_STP_, corrected F_ST_; F_IS_, inbreeding coefficient per overall loci; D_EST_, measure of population differentiation. Numbers in parentheses below each category indicate the average value.

The overall gene diversity (*H*_*T*_= 0.4644) and at the sub-population level (*H*_*s*_ = 0.462) are identical, which indicates that all populations mix freely and all the samples are essentially part of a single panmictic population (Figure 2). This is corroborated by a pairwise comparison between populations based on Nei's genetic distances (Supplementary Figure S3). F_ST_ measures the amount of genetic variance that can be explained by population structure based on Wright's F-statistics. A F_ST_ value of 0 indicates no differentiation between the subpopulations while a value of 1 indicates complete differentiation. The low genetic structure found shows that there is a relatively high gene flow (*F*_*ST*_ = 0.0051), which is consistent with the on-going exchange of seeds and plants in the region. The low fixation index within populations (*F*_*IS*_= −0.7944) and a lack of extensive differentiation among populations (*F*_*ST*_), is concordant with the low population differentiation (*D*_*ST*_= 0.0047).

### Population Structure

**Figure 3:**
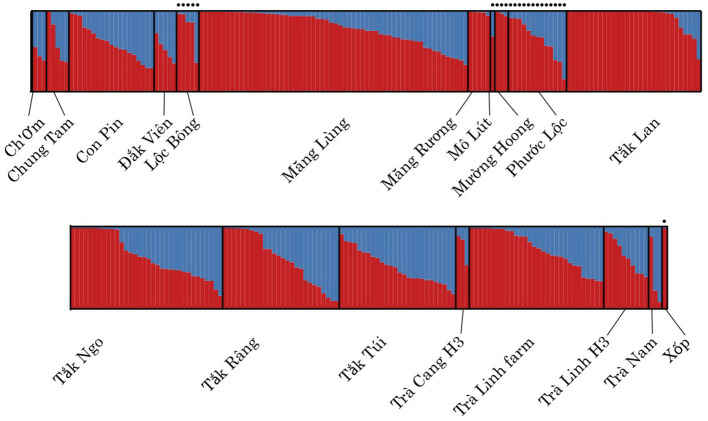
Admixture plots of *Panax vietnamensis* per population for K = 2. Columns topped with a black dot constitute wild samples. No consistent differences in the admixture patterns were detected between sites.

**Figure 4:**
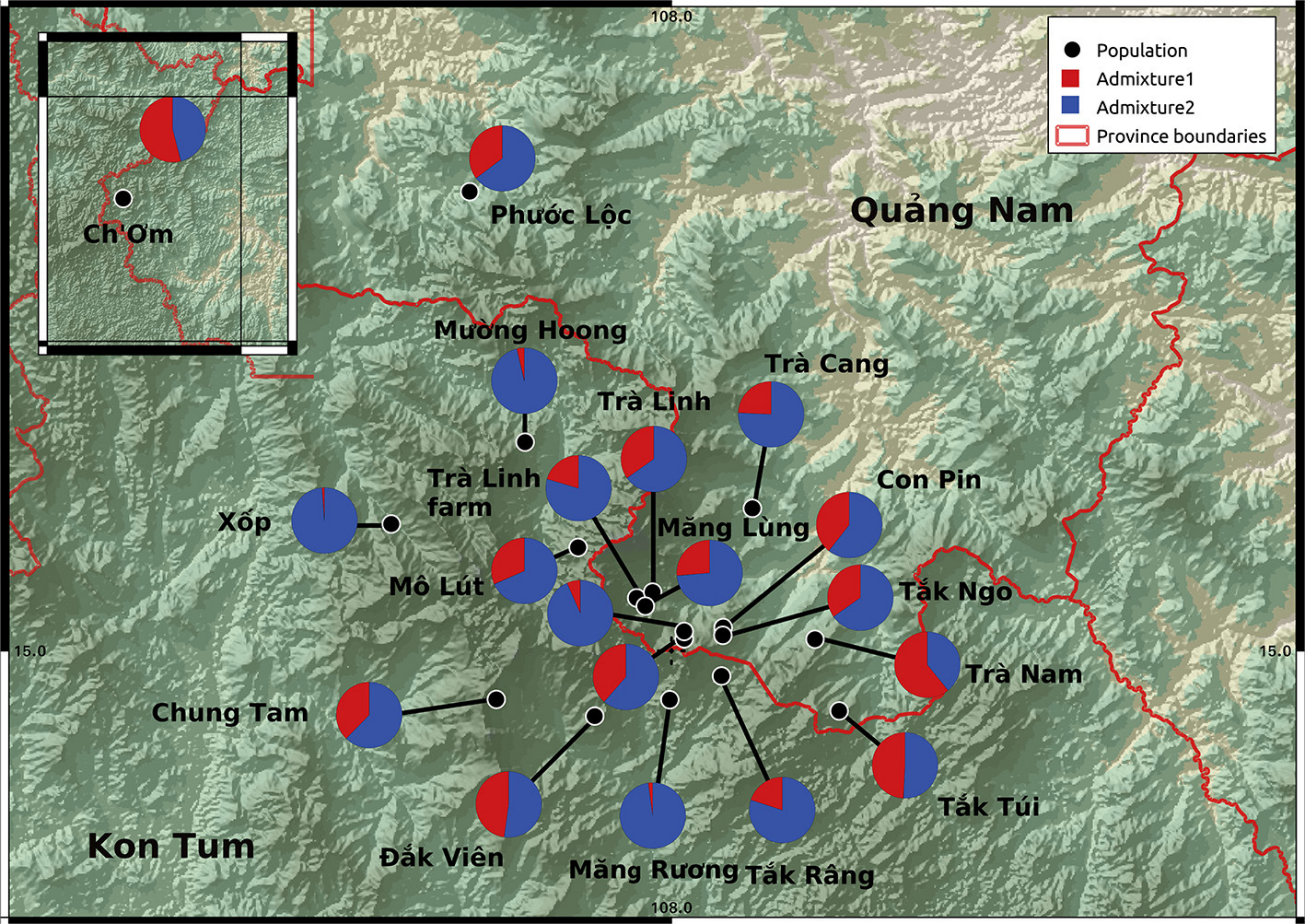
Map showing *Panax vietnamensis* population sampling locations with average admixture plots K = 2. No consistent differences in the admixture patterns were detected between sites.

The STRUCTURE analyses indicate that both wild and cultivated samples derive from the same two ancestral populations (Supplementary Figure S4). The STRUCTURE analysis showed a stabilization in the log likelihood value after *K* = 2 (Supplementary Figure S1), suggesting *K* = 2 has the optimal number of subpopulations identified within the genetic data also supported by the Δ*K* method (Supplementary Figures S1, S5). The admixture plot shows a wide range of admixture patterns among the individuals (Figure 3). Chung Tam, Măng Lùng, Măng R
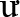

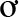
ng, M
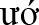
ng Hoong, T
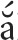
k Lan, T
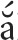
k Ngo, T
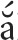
k Râng, and Trà Linh farm include admixed individuals and individuals from one ancestral population. Eleven populations present only admixted individuals with various proportions. This admixture pattern over the different populations suggests that the 19 populations did not show any distinct population genetic structure based on the dataset (Figure 4).

**Figure 5:**
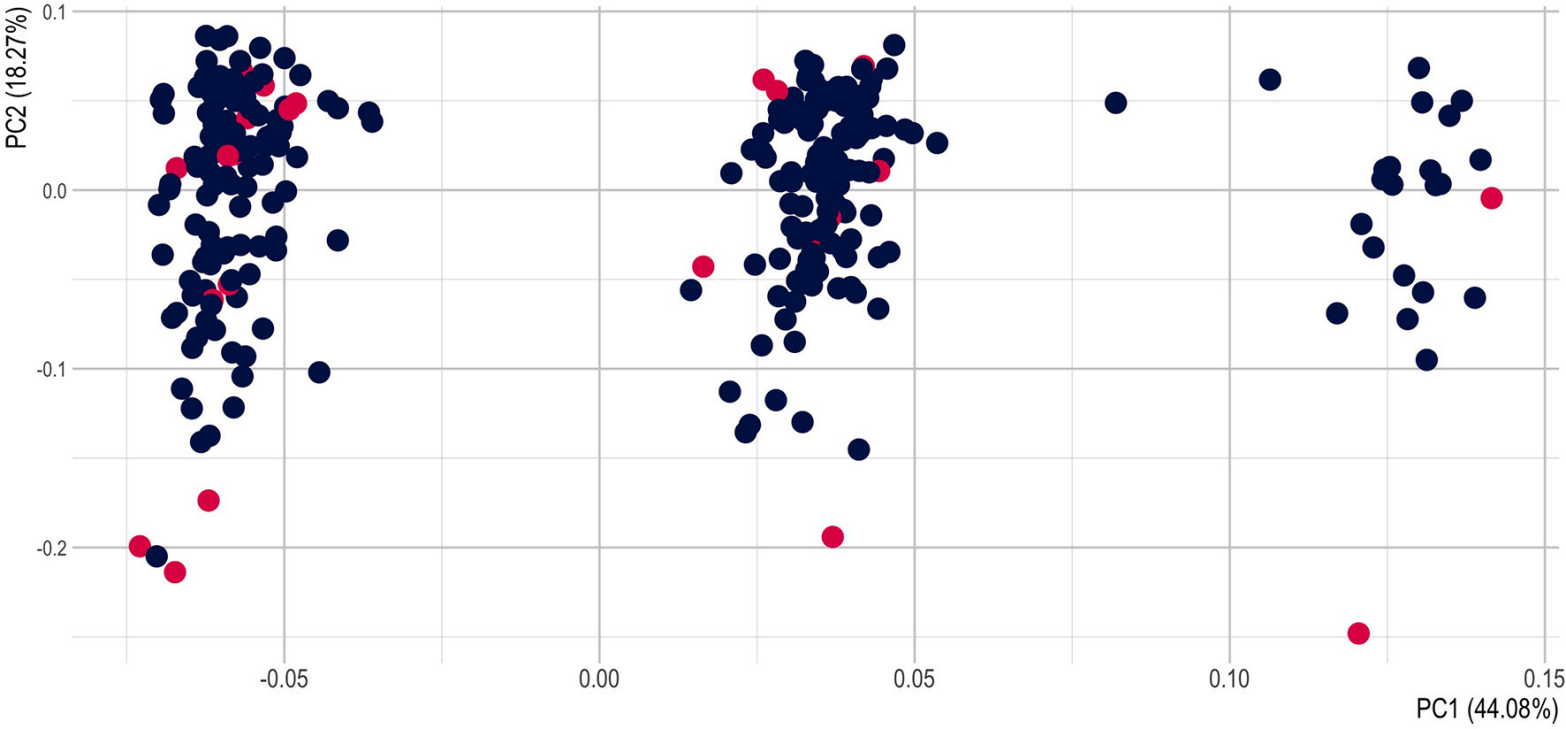
PCA of *Panax vietnamensis* populations showing wild (red) vs. cultivated (blue) samples.

The principal components account for a large proportion of the multidimensional variability, 59% for the PCA1 and PCA2 (Supplementary Figure S6). We plotted the principal components with the metadata (population, commune, district, province) and to the cultivation status (wild or cultivated) (Figure 5; Supplementary Figures S7–S9). We tested if there was a correlation between the metadata and the PCs but no PCs has a strong correlation with any of the metadata (Supplementary Figure S10), except for the Province and the PCA2.

The sub-sampled data sets show a high standard deviation on the likelihood estimates (Supplementary Material—SNP). The sub-sampling has reduced the number of SNPs and this introduces some stochasticity in the analysis. Despite these variations, all the sets converged to an optimal number of population of two (Supplementary Material and https://rpubs.com/vincentmanz/A). We compiled the average admixture value for each population. The results of the sub-sampling sets are highly consistent. The comparison of the averaged admixture proportions in the 19 populations between the sub-sampled and the full data sets, show that the admixture values of the randomized SNPs are not significantly different from the full data set (*p*-value = 0.6321). The presence of linked markers in our data set does not affect the STRUCTURE estimates.

## Discussion

The genetic diversity in the 1,181 SNP dataset is very high, supporting outcrossing of *P. vietnamensis*. The structure and population genomic analyses do not show any segregation between the 19 populations. Furthermore, there is no difference between cultivated individuals and those found in the wild. High genetic diversity and gene flow between *P. vietnamensis* populations have recently been attributed to insect dispersal (Vu et al., 2020). Strong gene flow and insufficient splitting time between cultivated populations has also been observed for *P. notoginseng* (Pan et al., 2016), and was attributed to non-random mating among the individuals of the population. Here we argue that high genetic diversity and the lack of differentiation between populations is due to extensive ancestral woodlot cultivation, management and exchange and trade practices.

Cultivated plants usually present profound morphological differences when compared to their parental populations, along with a reduced genetic diversity due to the bottleneck processes that occur during domestication (Doebley et al., 2006), yet this is not the case for *P. vietnamensis*. This could be the result of a short domestication history of *P. vietnamensis* combined with panmixia, or very weak genetic differentiation, or a continued cultivation understorey to meet consumer preference for wild materials (Liu et al., 2021). The high heterozygosity can be explained by the artificial gene flow among the population due to the trade between the farmers in the region. It is likely that the extensive pattern of admixture that we observe in both the wild and cultivated samples is due to anthropic influences, as observed for other plant species of high cultural value (Stefenon et al., 2008; Martínez-Castillo et al., 2014; Wiehle et al., 2014).

The little time depth of *P. vietnamensis* domestication history seems to result from a lack of deeper historical documentation rather than being indicative of a recent domestication process. While Vietnamese ginseng was only formally described in 1985, ginseng has been known for centuries in Vietnam. We can assume that ginseng was at least known from the fifteenth century when ambassadors from Vietnam in Beijing would have learnt about its value (Ock, 2019). The medicinal use of ginseng in neighboring China and more broadly in Southeast Asia predates this, and we can assume that this was also known in Vietnam. Ock proposes that Vietnamese-Korean trade as early as the nineteenth century would have brought Korean ginseng to Vietnam (Ock, 2019). Later, ginseng would have reached Vietnam as imperial gifts or through private trade from China, and from Ryukyu (islands between Japan and Taiwan), which were hubs of international trade (Ock, 2019). It is not possible to assert if local knowledge about the medicinal properties of *P. vietnamensis* predated the trade of ginseng from abroad. If local populations were aware of the properties of ginseng, trade would have facilitated the exploitation of *P. vietnamensis*. Knowledge of the medicinal use of ginseng would have traveled from the coast to the mountains and local populations there, and awareness that similar species occurred naturally in that area, people would have started harvesting and trading these species. Eventually, transplanting young individuals to villages or local forests provided better control of the resource as it matured (Koh, 2019). Koh (2019) concludes that as cultivation of ginseng expanded, wild ginseng harvesting decreased. Yet, it might be that the expansion of cultivation included woodlot or understorey cultivation, and the high degrees of management of wild populations present today could result in the observed lack of differentiation among these. Alternatively, the occurrence of Vietnamese ginseng might not have been known to either local ethnic minorities or people living along the coast. In that case either the coastal trade in ginseng did not extend to the mountain areas or no link was made between traded ginseng material and the local wild populations of Vietnamese ginseng. Nevertheless, local ethnic minorities living in the Annamite mountains might have been using Vietnamese ginseng themselves, and transplanting and mixing populations in the process. For instance, Vietnamese ginseng had long been used by the local X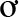 Ðăng ethnic group living at the foot of Mount Ngȯc Linh.

Given the migration history of the region, with many ethnic minorities in Vietnam having migrated from or through China, it is possible that ginseng cultivation and use traditions had been picked up and brought by migrants. Despite the fact that *P. vietnamensis* is mostly found in the high mountain area of central Vietnam, it is possible that migrants brought *P. vietnamensis* with them from China and that *P. vietnamensis* is in fact a selected variety of *P. japonica*. For instance, *P. vietnamensis* var. *fuscidiscus* is found in northern Vietnam. Further research is needed to fully understand if people's migratory history shaped the evolution and distribution of *P. vietnamensis*. In the absence of written historical records, a cross-cultural, comparative review of names and uses from all these ethnic minorities (Teixidor-Toneu et al., 2018) could provide further evidence of the history of ginseng presence and cultural use in the region.

The blurred distinction we observe here between wild and cultivated *P. vietnamensis* samples highlights the importance of participatory conservation approaches that fully integrate local knowledge and practice. The continued development of ginseng forest farming is desirable as a sustainable strategy to achieve demands for wild produce while preserving the species wild populations (Stefenon et al., 2008). Strategies that are co-designed between scientists and indigenous peoples and local communities can successfully and ethically conserve culturally important species (Sterling et al., 2017) and are better adapted to local conditions (Maldonado et al., 2016).

## Conclusions

The population structure of a total of 319 individuals of *Panax vietnamensis* was analyzed based on 1,181 SNP markers. Our study shows that all known *P. vietnamensis* populations, wild and cultivated, are part of one single panmictic population and cannot be differentiated genetically. Both wild and cultivated samples derive from two ancestral populations and show varying degrees of admixture. Given the continuum of cultivation practices from fertilized and irrigated fields in specialized cultivation centers and small-scale farms to understorey untended transplants that are later harvested, and the exchange and trade activities throughout the region, we attribute the observed high genetic diversity and gene flow to anthropic influence. These results showcase the importance of integrating and understanding local ecological knowledge and practice for the sustainable management of culturally valuable plants.

## Data Availability Statement

The original contributions presented in the study are publicly available. This data can be found here: National Center for Biotechnology Information (NCBI) BioProject database under Accession Number PRJNA788747.

## Author Contributions

HTTL, HVN, HJDB, and VM conceived and initiated the project and designed the experimental study. LNN, HLPB, HTML, TDL, and VTN collected data. LNN and VM analyzed data. VM, LNN, HTTL, and HJDB interpreted results. HD, ITT, HTTL, LNN, and VM wrote the article. All authors read, commented, and approved the final manuscript version.

## Funding

This work was performed on the High Performance Computer (HPC) system in the Institute of Genome Research, Vietnam Academy of Science and Technology, Vietnam and on resources provided by UNINETT Sigma2—The National Infrastructure for High Performance Computing and Data Storage in Norway. This project was supported by the Ministry of Science and Technology, Vietnam under the project Transcriptome Sequencing and Analysis of *Panax vietnamensis* Ha and Grushv. (Grant No. 16/2017-HÐ-NVQG) and the European Union's Seventh Framework Program for Research, Technological Development and Demonstration under the Grant Agreement No. 606895 to the FP7-MCA-ITN MedPlant, Phylogenetic exploration of medicinal plant diversity. This project has received funding from the European Union's Horizon 2020 research and innovation program under the Marie Skłodowska-Curie Grant Agreement No. 841127.

## Conflict of Interest

VM was employed by Baseclear BV. The remaining authors declare that the research was conducted in the absence of any commercial or financial relationships that could be construed as a potential conflict of interest.

## Publisher's Note

All claims expressed in this article are solely those of the authors and do not necessarily represent those of their affiliated organizations, or those of the publisher, the editors and the reviewers. Any product that may be evaluated in this article, or claim that may be made by its manufacturer, is not guaranteed or endorsed by the publisher.

## Supplementary Material

Supplementary Figure S1 The estimated ln probability of data given the *K-*value. Error bars are standard deviations of 3 replicate runs.

Supplementary Figure S2 Heat map of Nei's genetic distances showing the average number of pairwise differences between populations.

Supplementary Figure S3 Coverage of the markers per sample.

Supplementary Figure S4 Pair plot PCAs.

Supplementary Figure S5 PCAs with individuals colored by populations.

Supplementary Figure S6 PCAs with individuals colored by HiSeq lane.

Supplementary Figure S7 PCAs with individuals colored by province.

Supplementary Figure S8 Pearson *r*^2^ correlates of the principal components back to the metadata. The asterisk symbol indicates significant correlation of the metadata on the specific PCA axis.

Supplementary Figure S9 Admixture plots for k equal two to eight.

Supplementary Figure S10 Admixture plots for *K* = 2 sorted by samples of wild vs. cultivated origin.

Supplementary Table S1 Voucher specimen information.

Supplementary Table S2 Populations' information.
