# Supplementary figures and images for: Target Capture Reveals the Complex Origin of Vietnamese Ginseng
Source: Front Plant Sci. 2022 Jul 13;13:814178. doi: 10.3389/fpls.2022.814178 (PMC9326450; doi:10.3389/fpls.2022.814178)

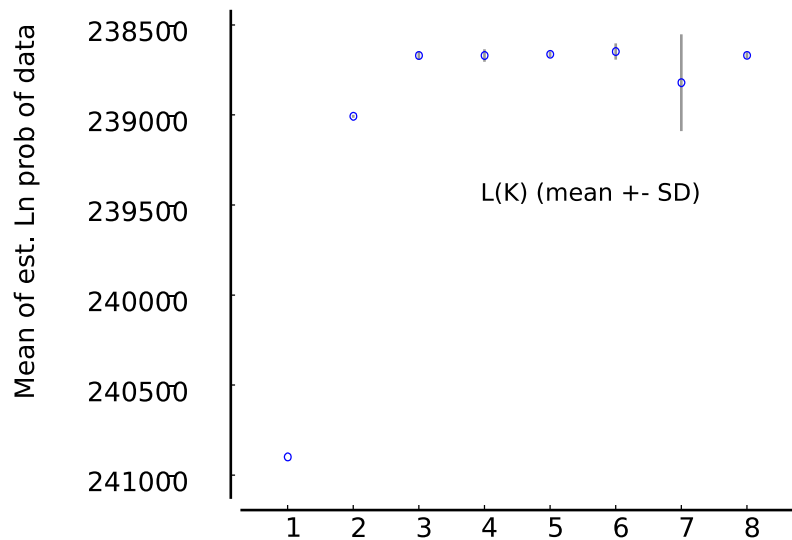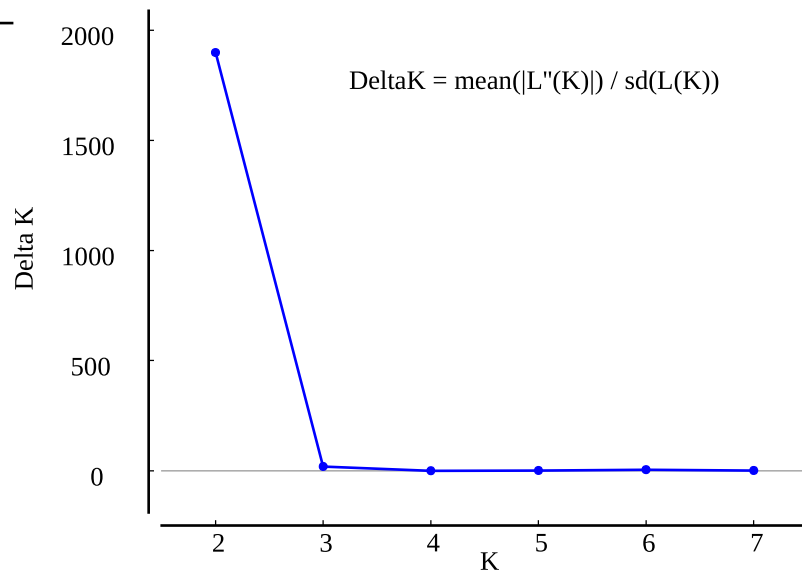

Supplement: Supplementary Figure S1 — The estimated ln probability of data given the K-value. Error bars are standard deviations of 3 replicate runs. [file Image_1.PDF]

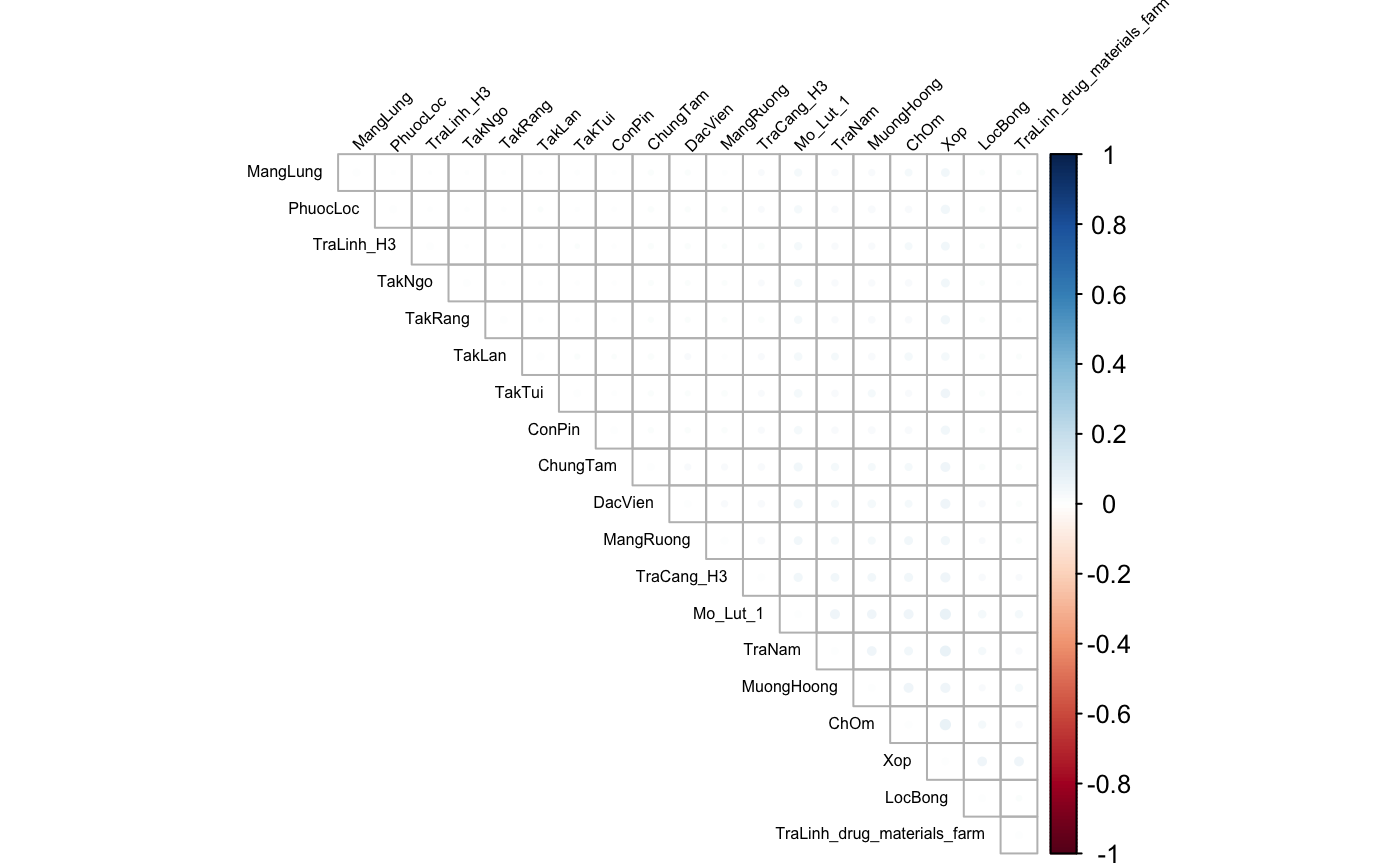

Supplement: Supplementary Figure S2 — Heat map of Nei's genetic distances showing the average number of pairwise differences between populations. [file Image_2.PNG]

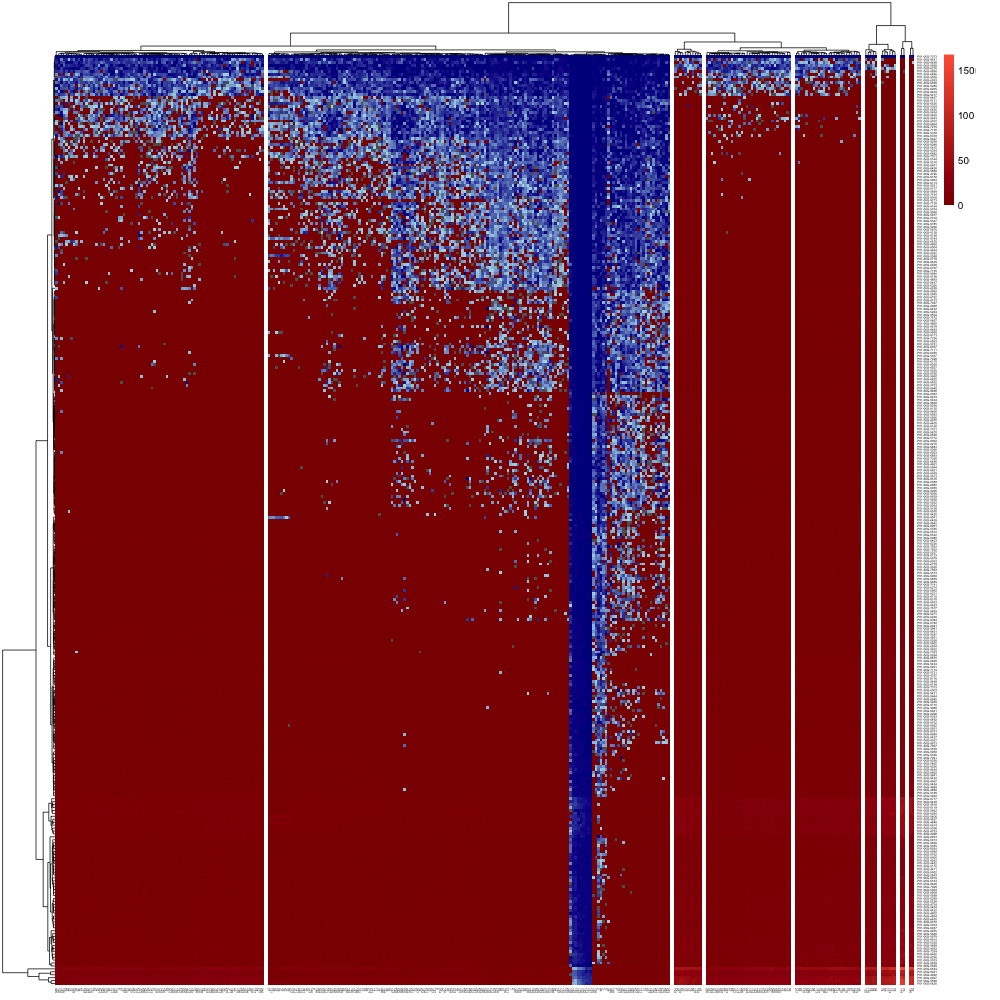

Supplement: Supplementary Figure S3 — Coverage of the markers per sample. [file Image_3.PNG]

**PC1,  
44.08%**

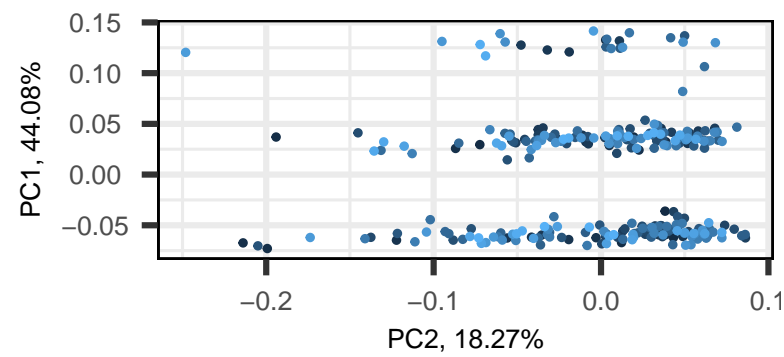

**PC2,  
18.27%**

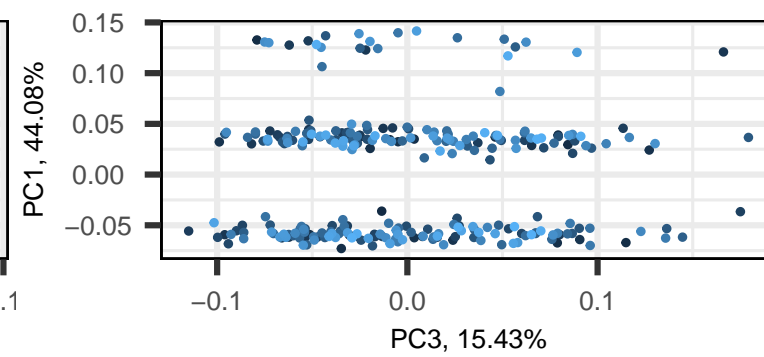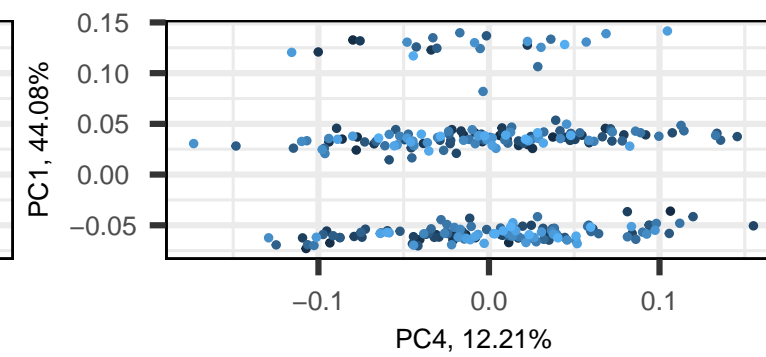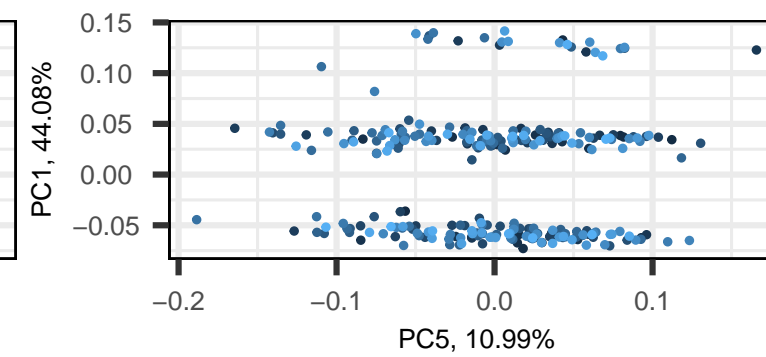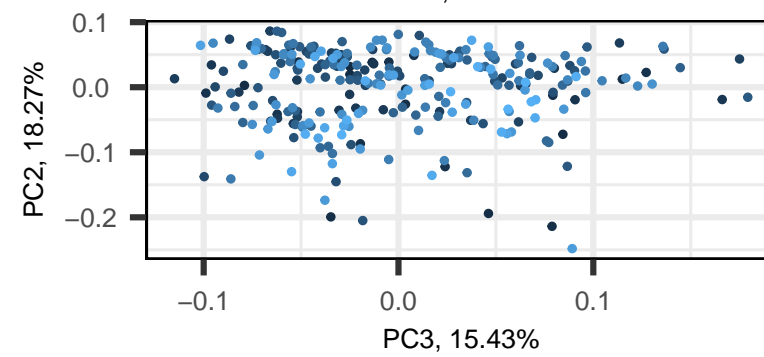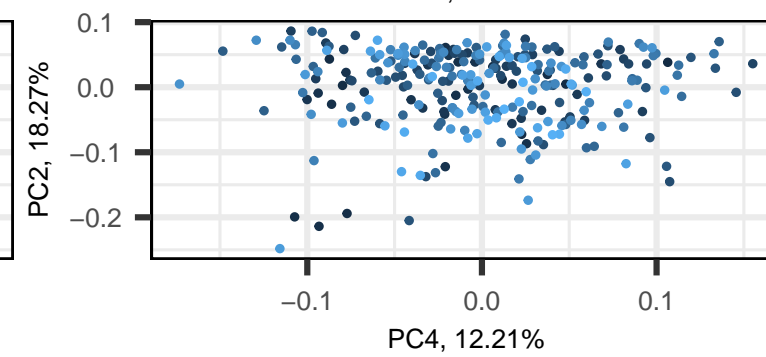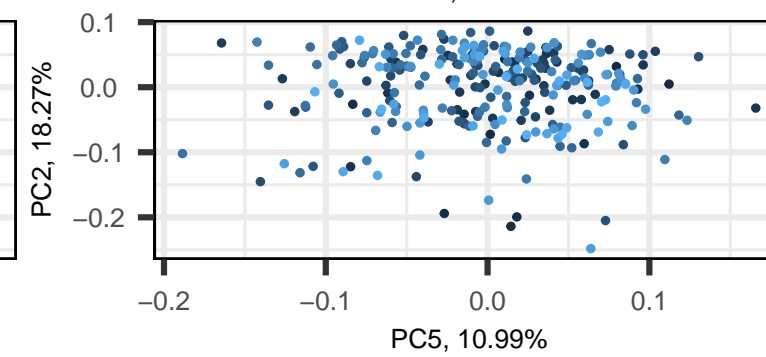

**PC3,  
15.43%**

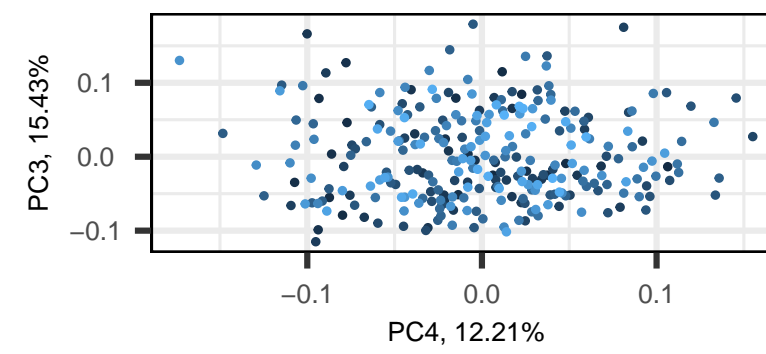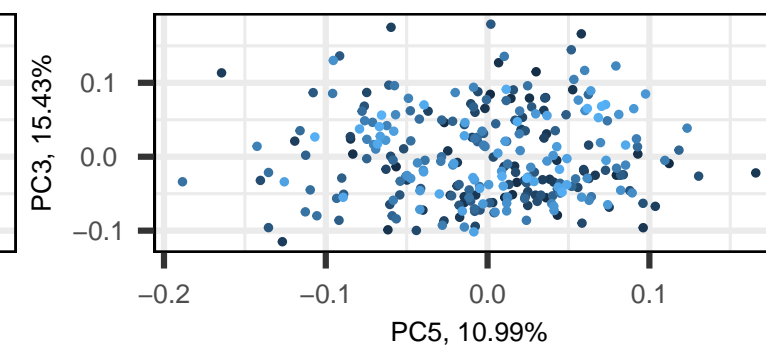

**PC4,  
12.21%**

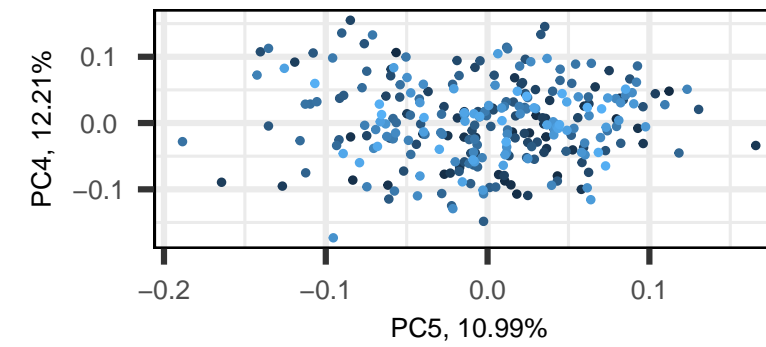

**PC5,  
10.99%**

Supplement: Supplementary Figure S4 — Pair plot PCAs. [file Image_4.PDF]

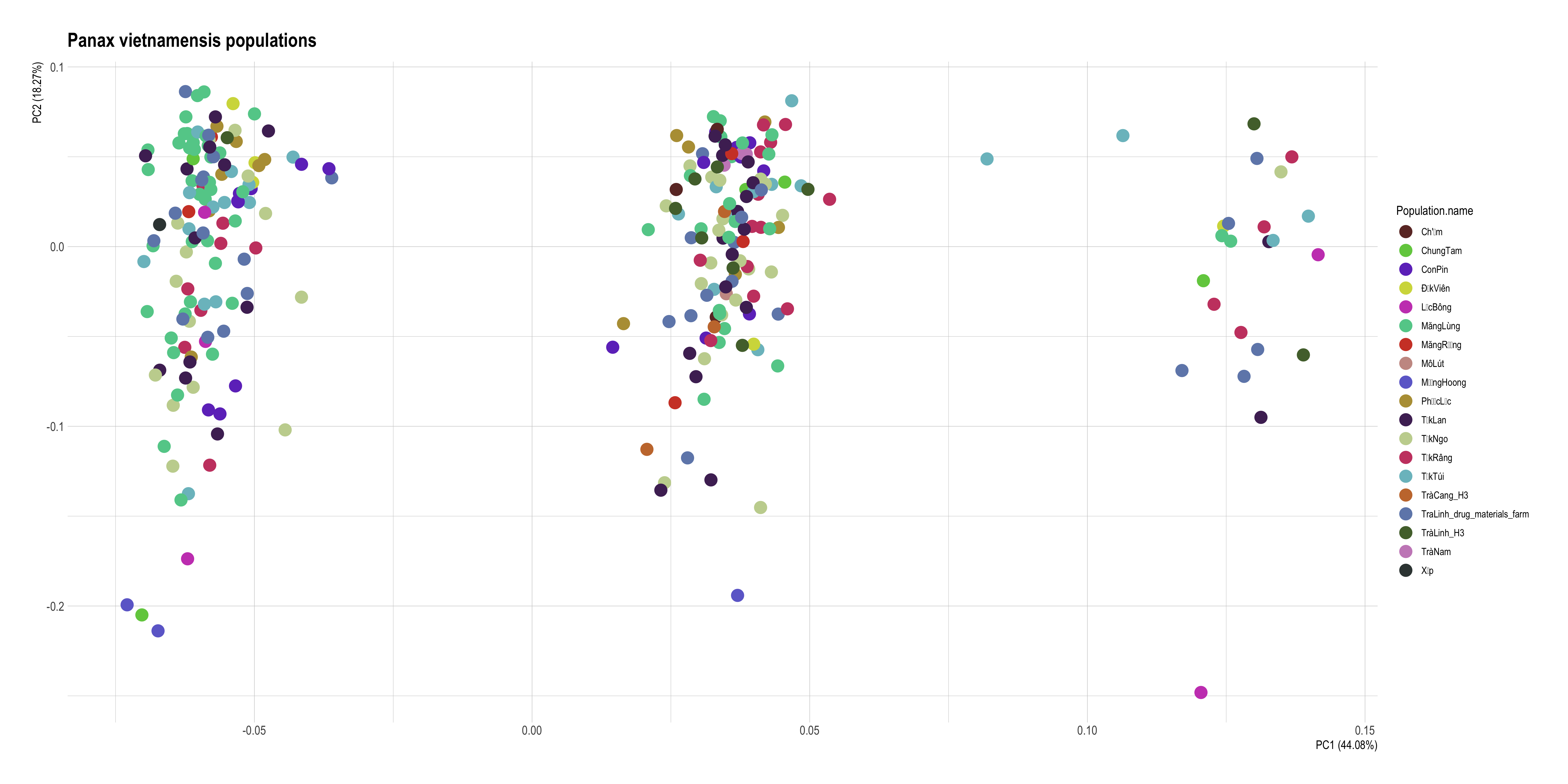

Supplement: Supplementary Figure S5 — PCAs with individuals colored by populations. [file Image_5.PNG]

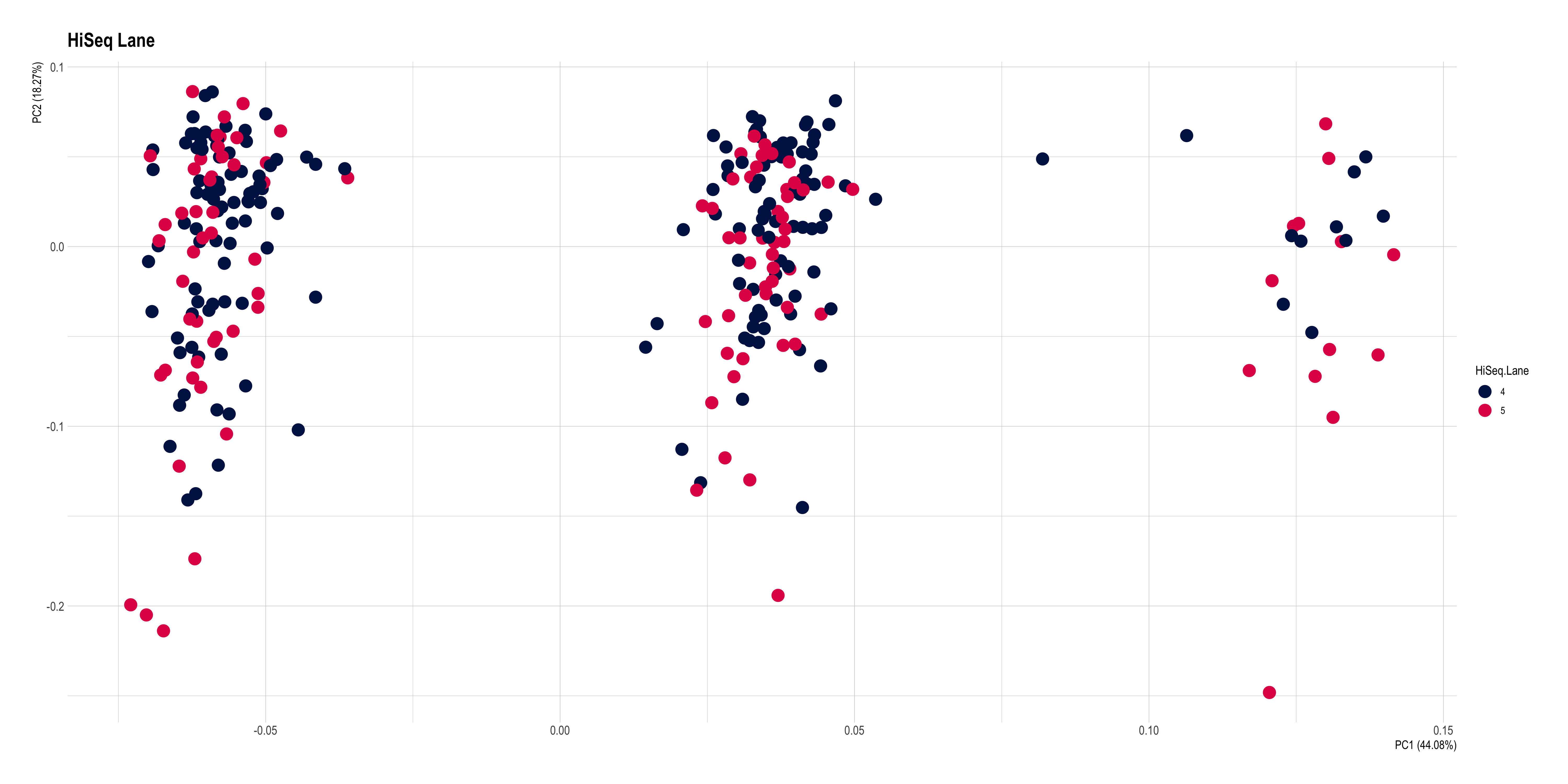

Supplement: Supplementary Figure S6 — PCAs with individuals colored by HiSeq lane. [file Image_6.PNG]

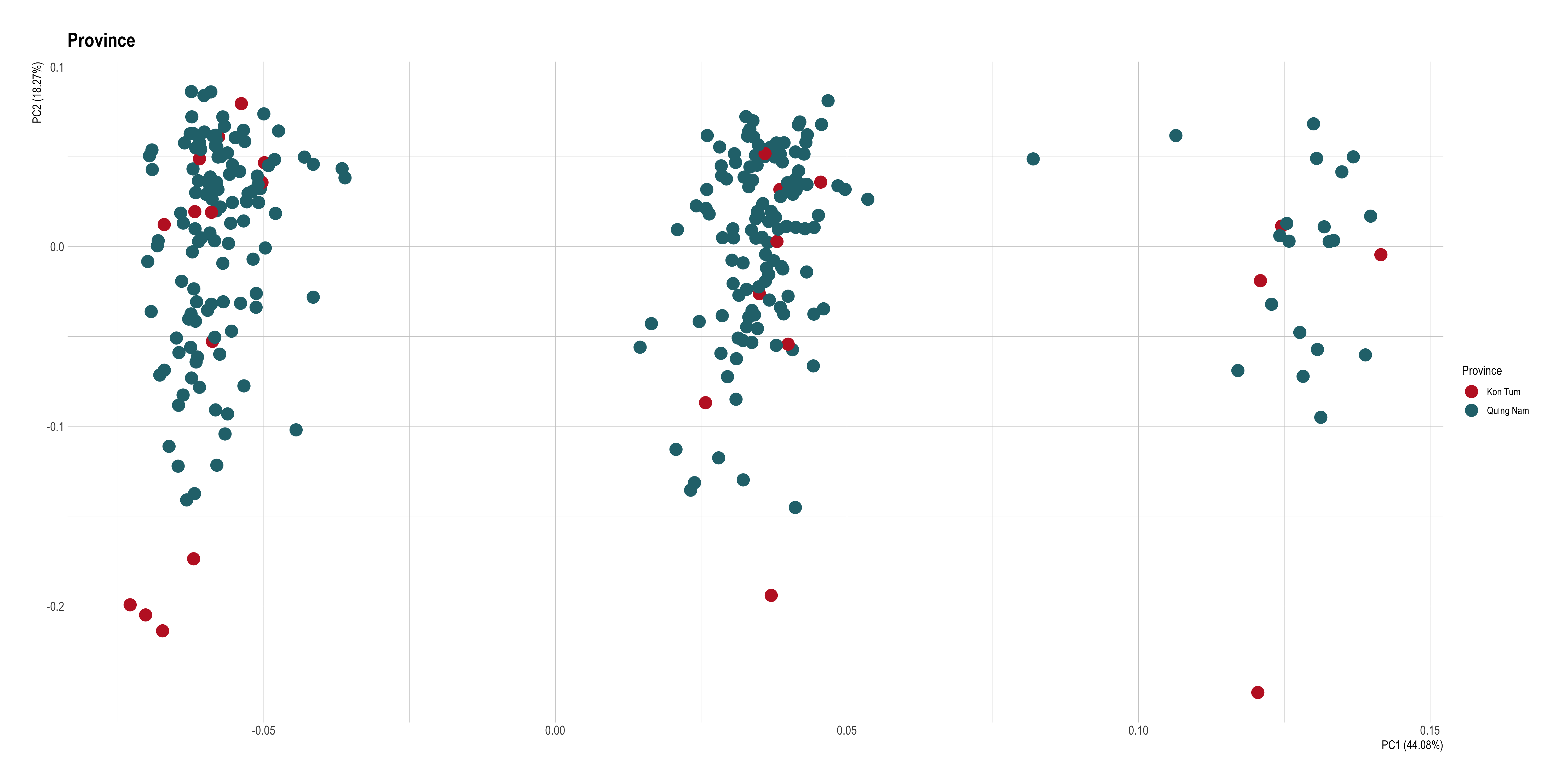

Supplement: Supplementary Figure S7 — PCAs with individuals colored by province. [file Image_7.PNG]

Principal component Pearson  $r^2$  metadata correlates

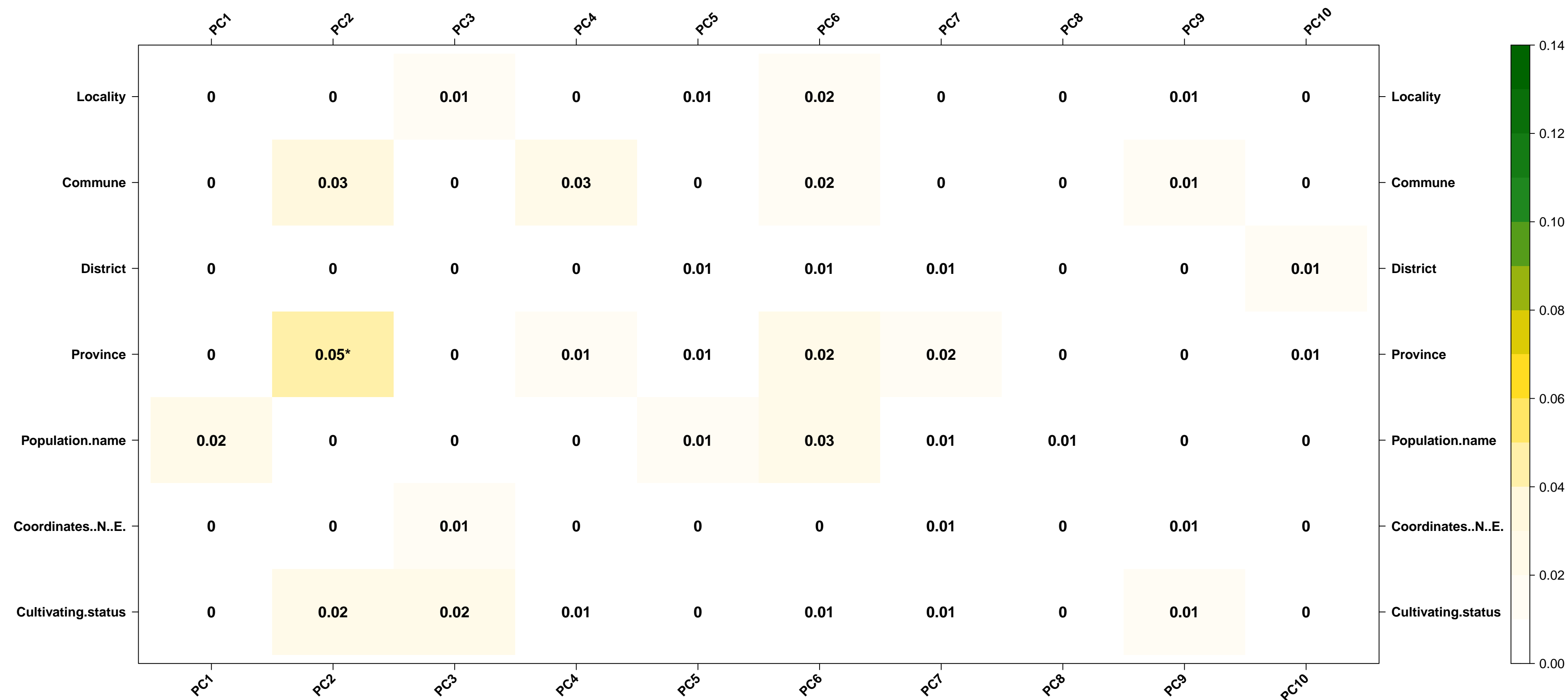

Supplement: Supplementary Figure S8 — Pearson r2 correlates of the principal components back to the metadata. The asterisk symbol indicates significant correlation of the metadata on the specific PCA axis. [file Image_8.PDF]

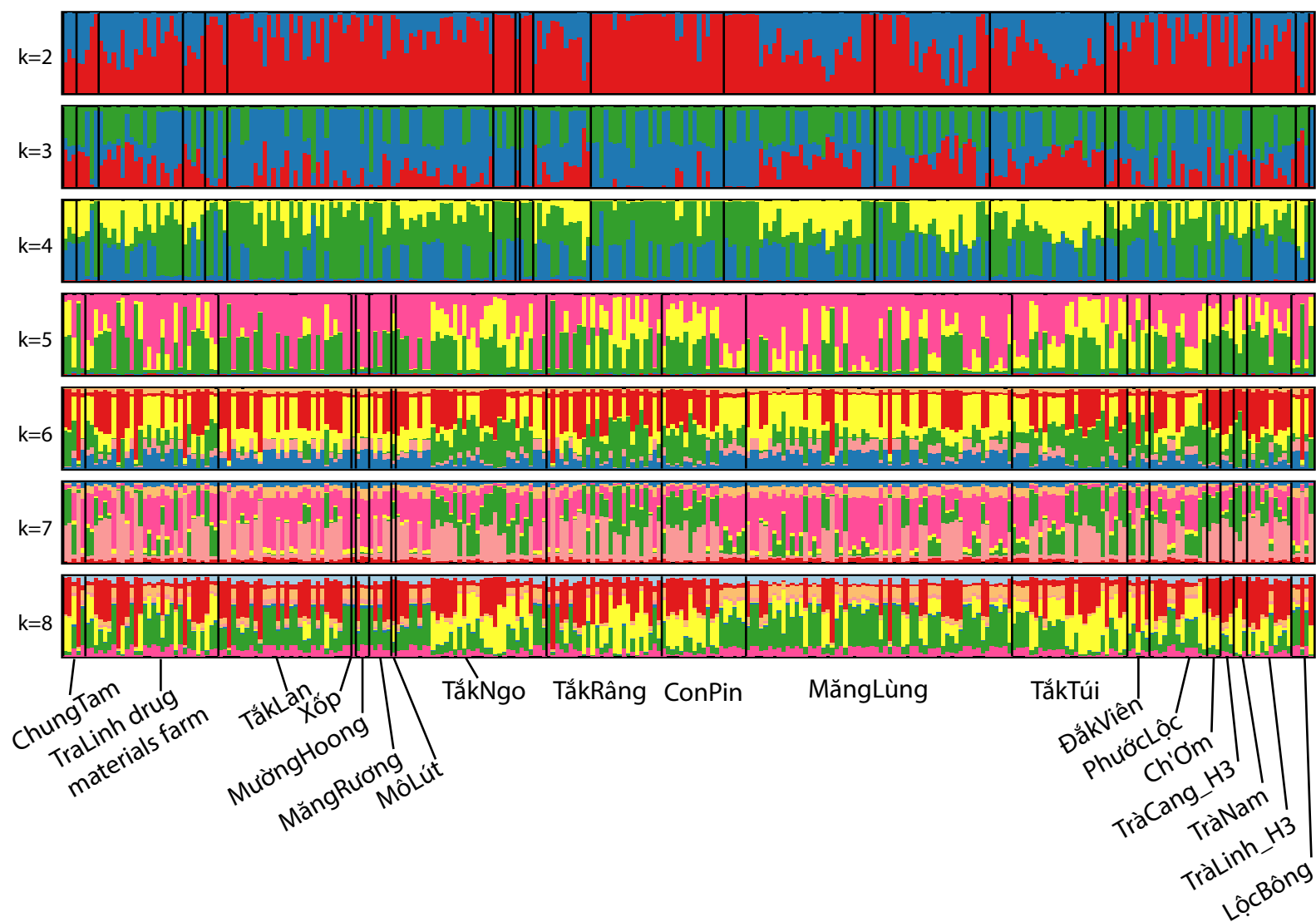

Supplement: Supplementary Figure S9 — Admixture plots for k equal two to eight. [file Image_9.PDF]

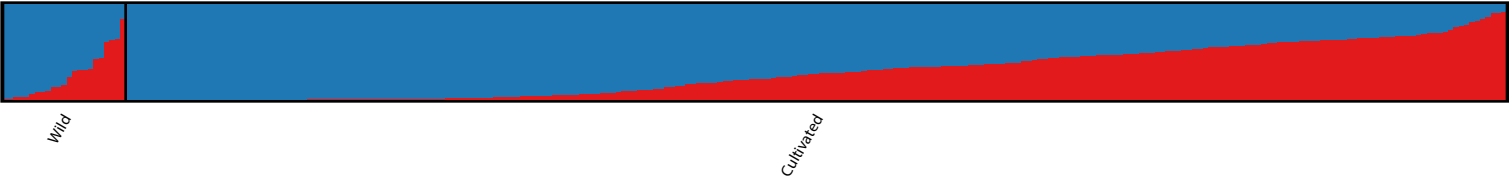

Supplement: Supplementary Figure S10 — Admixture plots for K = 2 sorted by samples of wild vs. cultivated origin. [file Image_10.PDF]
