# Supplementary material for: Target Capture Reveals the Complex Origin of Vietnamese Ginseng
Source: Front Plant Sci. 2022 Jul 13;13:814178. doi: 10.3389/fpls.2022.814178 (PMC9326450; doi:10.3389/fpls.2022.814178)
Supplement: Supplementary Table S1 — Voucher specimen information. [file Data_Sheet_1.PDF]

Population name	Sample No.	Sample name	Sample ID	Province	District	Commune	Locality	Coordinates (N, E)	Altitude (m)	Cultivating status
ĐắkViên	6	TX6	17	Kon Tum	Tu Mơ Rông	Tê Xăng	Đắk Viên	14.9610, 107.9540	1722	Cultivated
ĐắkViên	5	TX5	21	Kon Tum	Tu Mơ Rông	Tê Xăng	Đắk Viên	14.9610, 107.9540	1722	Cultivated
ĐắkViên	12	TX12	132	Kon Tum	Tu Mơ Rông	Tê Xăng	Đắk Viên	14.9610, 107.9540	1722	Cultivated
ĐắkViên	11	TX11	133	Kon Tum	Tu Mơ Rông	Tê Xăng	Đắk Viên	14.9610, 107.9540	1722	Cultivated
ĐắkViên	8	TX8	134	Kon Tum	Tu Mơ Rông	Tê Xăng	Đắk Viên	14.9610, 107.9540	1722	Cultivated
ĐắkViên	13	TX13	135	Kon Tum	Tu Mơ Rông	Tê Xăng	Đắk Viên	14.9610, 107.9540	1722	Cultivated
ĐắkViên	10	TX10	136	Kon Tum	Tu Mơ Rông	Tê Xăng	Đắk Viên	14.9610, 107.9540	1722	Cultivated
ĐắkViên	9	TX9	138	Kon Tum	Tu Mơ Rông	Tê Xăng	Đắk Viên	14.9610, 107.9540	1722	Cultivated
ĐắkViên	4	TX4	18	Kon Tum	Tu Mơ Rông	Tê Xăng	Đắk Viên	14.9610, 107.9540	1722	Cultivated
ĐắkViên	3	TX3	19	Kon Tum	Tu Mơ Rông	Tê Xăng	Đắk Viên	14.9610, 107.9540	1722	Cultivated
ĐắkViên	2	TX2	22	Kon Tum	Tu Mơ Rông	Tê Xăng	Đắk Viên	14.9610, 107.9540	1722	Cultivated
ĐắkViên	7	TX7	23	Kon Tum	Tu Mơ Rông	Tê Xăng	Đắk Viên	14.9610, 107.9540	1722	Cultivated
ĐắkViên	1	TX1	24	Kon Tum	Tu Mơ Rông	Tê Xăng	Đắk Viên	14.9610, 107.9540	1722	Cultivated
TắkTúi	27	TT24	231	Quảng Nam	Nam Trà My	Trà Nam	Tắk Túi	14.9641 108.1005	1623	Cultivated
TắkTúi	23	TT20	234	Quảng Nam	Nam Trà My	Trà Nam	Tắk Túi	14.9641 108.1005	1623	Cultivated
TắkTúi	11	TT8	263	Quảng Nam	Nam Trà My	Trà Nam	Tắk Túi	14.9641 108.1005	1623	Cultivated
TắkTúi	33	TT30	282	Quảng Nam	Nam Trà My	Trà Nam	Tắk Túi	14.9641 108.1005	1623	Cultivated
TắkTúi	20	TT17	179	Quảng Nam	Nam Trà My	Trà Nam	Tắk Túi	14.9641 108.1005	1623	Cultivated
TắkTúi	18	TT15	180	Quảng Nam	Nam Trà My	Trà Nam	Tắk Túi	14.9641 108.1005	1623	Cultivated
TắkTúi	17	TT14	181	Quảng Nam	Nam Trà My	Trà Nam	Tắk Túi	14.9641 108.1005	1623	Cultivated
TắkTúi	14	TT11	182	Quảng Nam	Nam Trà My	Trà Nam	Tắk Túi	14.9641 108.1005	1623	Cultivated
TắkTúi	19	TT16	183	Quảng Nam	Nam Trà My	Trà Nam	Tắk Túi	14.9641 108.1005	1623	Cultivated
TắkTúi	16	TT13	184	Quảng Nam	Nam Trà My	Trà Nam	Tắk Túi	14.9641 108.1005	1623	Cultivated
TắkTúi	21	TT18	185	Quảng Nam	Nam Trà My	Trà Nam	Tắk Túi	14.9641 108.1005	1623	Cultivated
TắkTúi	15	TT12	186	Quảng Nam	Nam Trà My	Trà Nam	Tắk Túi	14.9641 108.1005	1623	Cultivated
TắkTúi	28	TT25	227	Quảng Nam	Nam Trà My	Trà Nam	Tắk Túi	14.9641 108.1005	1623	Cultivated
TắkTúi	26	TT23	228	Quảng Nam	Nam Trà My	Trà Nam	Tắk Túi	14.9641 108.1005	1623	Cultivated
TắkTúi	25	TT22	229	Quảng Nam	Nam Trà My	Trà Nam	Tắk Túi	14.9641 108.1005	1623	Cultivated
TắkTúi	22	TT19	230	Quảng Nam	Nam Trà My	Trà Nam	Tắk Túi	14.9641 108.1005	1623	Cultivated
TắkTúi	24	TT21	232	Quảng Nam	Nam Trà My	Trà Nam	Tắk Túi	14.9641 108.1005	1623	Cultivated
TắkTúi	29	TT26	233	Quảng Nam	Nam Trà My	Trà Nam	Tắk Túi	14.9641 108.1005	1623	Cultivated
TắkTúi	12	TT9	259	Quảng Nam	Nam Trà My	Trà Nam	Tắk Túi	14.9641 108.1005	1623	Cultivated
TắkTúi	10	TT7	260	Quảng Nam	Nam Trà My	Trà Nam	Tắk Túi	14.9641 108.1005	1623	Cultivated
TắkTúi	9	TT6	261	Quảng Nam	Nam Trà My	Trà Nam	Tắk Túi	14.9641 108.1005	1623	Cultivated
TắkTúi	6	TT3	262	Quảng Nam	Nam Trà My	Trà Nam	Tắk Túi	14.9641 108.1005	1623	Cultivated
TắkTúi	8	TT5	264	Quảng Nam	Nam Trà My	Trà Nam	Tắk Túi	14.9641 108.1005	1623	Cultivated
TắkTúi	13	TT10	265	Quảng Nam	Nam Trà My	Trà Nam	Tắk Túi	14.9641 108.1005	1623	Cultivated

TắkTúi	7	TT4	266	Quảng Nam	Nam Trà My	Trà Nam	Tắk Túi	14.9641 108.1005	1623	Cultivated
TắkTúi	32	TT29	278	Quảng Nam	Nam Trà My	Trà Nam	Tắk Túi	14.9641 108.1005	1623	Cultivated
TắkTúi	5	TT2	289	Quảng Nam	Nam Trà My	Trà Nam	Tắk Túi	14.9641 108.1005	1623	Cultivated
TắkTúi	31	TT28	299	Quảng Nam	Nam Trà My	Trà Nam	Tắk Túi	14.9641 108.1005	1623	Cultivated
TắkTúi	4	TT1	300	Quảng Nam	Nam Trà My	Trà Nam	Tắk Túi	14.9641 108.1005	1623	Cultivated
TắkTúi	30	TT27	303	Quảng Nam	Nam Trà My	Trà Nam	Tắk Túi	14.9641 108.1005	1623	Cultivated
MăngRương	2	MRương2	57	Kon Tum	Tu Mơ Rông	Ngọk Lây	Măng Rương	14.9708, 107.9990	1555	Cultivated
MăngRương	1	MRương1	61	Kon Tum	Tu Mơ Rông	Ngọk Lây	Măng Rương	14.9708, 107.9990	1555	Cultivated
MăngRương	3	MRương3	63	Kon Tum	Tu Mơ Rông	Ngọk Lây	Măng Rương	14.9708, 107.9990	1555	Cultivated
MăngRương	4	MRương4	108	Kon Tum	Tu Mơ Rông	Ngọk Lây	Măng Rương	14.9708, 107.9990	1555	Cultivated
MăngRương	5	MRương5	112	Kon Tum	Tu Mơ Rông	Ngọk Lây	Măng Rương	14.9708, 107.9990	1555	Cultivated
ChungTam	2	MR2	2	Kon Tum	Tu Mơ Rông	Mang Ri	Chung Tam	14.9710, 107.8950	2008	Cultivated
ChungTam	1	MR1	3	Kon Tum	Tu Mơ Rông	Mang Ri	Chung Tam	14.9710, 107.8950	2008	Cultivated
ChungTam	3	MR3	5	Kon Tum	Tu Mơ Rông	Mang Ri	Chung Tam	14.9710, 107.8950	2008	Cultivated
ChungTam	5	MR5	7	Kon Tum	Tu Mơ Rông	Mang Ri	Chung Tam	14.9710, 107.8950	2008	Cultivated
ChungTam	12	MR12	123	Kon Tum	Tu Mơ Rông	Mang Ri	Chung Tam	14.9710, 107.8950	2008	Cultivated
ChungTam	10	MR10	124	Kon Tum	Tu Mơ Rông	Mang Ri	Chung Tam	14.9710, 107.8950	2008	Cultivated
ChungTam	9	MR9	125	Kon Tum	Tu Mơ Rông	Mang Ri	Chung Tam	14.9710, 107.8950	2008	Cultivated
ChungTam	8	MR8	128	Kon Tum	Tu Mơ Rông	Mang Ri	Chung Tam	14.9710, 107.8950	2008	Cultivated
ChungTam	7	MR7	130	Kon Tum	Tu Mơ Rông	Mang Ri	Chung Tam	14.9710, 107.8950	2008	Cultivated
ChungTam	4	MR4	1	Kon Tum	Tu Mơ Rông	Mang Ri	Chung Tam	14.9710, 107.8950	2008	Cultivated
ChungTam	14	MR14	20	Kon Tum	Tu Mơ Rông	Mang Ri	Chung Tam	14.9710, 107.8950	2008	Cultivated
ChungTam	6	MR6	126	Kon Tum	Tu Mơ Rông	Mang Ri	Chung Tam	14.9710, 107.8950	2008	Cultivated
ChungTam	11	MR11	127	Kon Tum	Tu Mơ Rông	Mang Ri	Chung Tam	14.9710, 107.8950	2008	Cultivated
ChungTam	13	MR13	129	Kon Tum	Tu Mơ Rông	Mang Ri	Chung Tam	14.9710, 107.8950	2008	Cultivated
LộcBông	1	NLây1	131	Kon Tum	Tu Mơ Rông	Ngọk Lây	Lộc Bông	14.9850, 108.0300	781	Wild
LộcBông	2	NLây2	137	Kon Tum	Tu Mơ Rông	Ngọk Lây	Lộc Bông	14.9850, 108.0301	781	Wild
LộcBông	7	NLây7	58	Kon Tum	Tu Mơ Rông	Ngọk Lây	Lộc Bông	14.9850, 108.0302	781	Wild
LộcBông	6	NLây6	59	Kon Tum	Tu Mơ Rông	Ngọk Lây	Lộc Bông	14.9850, 108.0303	781	Wild
LộcBông	3	NLây3	60	Kon Tum	Tu Mơ Rông	Ngọk Lây	Lộc Bông	14.9850, 108.0304	781	Wild
LộcBông	5	NLây5	62	Kon Tum	Tu Mơ Rông	Ngọk Lây	Lộc Bông	14.9850, 108.0305	781	Wild
LộcBông	4	NLây4	64	Kon Tum	Tu Mơ Rông	Ngọk Lây	Lộc Bông	14.9850, 108.0306	781	Wild
TràNam	3	Trà Nam 3	283	Quảng Nam	Nam Trà My	Trà Nam		15.0070, 108.0860	1362	Cultivated
TràNam	1	Trà Nam 1	287	Quảng Nam	Nam Trà My	Trà Nam		15.0070, 108.0860	1362	Cultivated
TràNam	2	Trà Nam 2	301	Quảng Nam	Nam Trà My	Trà Nam		15.0070, 108.0860	1362	Cultivated
TắkRâng	14	TR10	151	Quảng Nam	Nam Trà My	Trà Cang	Tắk Râng	15.0073, 108.0076	1785	Cultivated
TắkRâng	10	TR6	154	Quảng Nam	Nam Trà My	Trà Cang	Tắk Râng	15.0073, 108.0076	1785	Cultivated
TắkRâng	5	TR1	276	Quảng Nam	Nam Trà My	Trà Cang	Tắk Râng	15.0073, 108.0076	1785	Cultivated

TắkRâng	6	TR2	279	Quảng Nam	Nam Trà My	Trà Cang	Tắk Râng	15.0073, 108.0076	1785	Cultivated
TắkRâng	8	TR4	281	Quảng Nam	Nam Trà My	Trà Cang	Tắk Râng	15.0073, 108.0076	1785	Cultivated
TắkRâng	31	TR27	139	Quảng Nam	Nam Trà My	Trà Cang	Tắk Râng	15.0073, 108.0076	1785	Cultivated
TắkRâng	29	TR25	140	Quảng Nam	Nam Trà My	Trà Cang	Tắk Râng	15.0073, 108.0076	1785	Cultivated
TắkRâng	28	TR24	141	Quảng Nam	Nam Trà My	Trà Cang	Tắk Râng	15.0073, 108.0076	1785	Cultivated
TắkRâng	25	TR21	142	Quảng Nam	Nam Trà My	Trà Cang	Tắk Râng	15.0073, 108.0076	1785	Cultivated
TắkRâng	30	TR26	143	Quảng Nam	Nam Trà My	Trà Cang	Tắk Râng	15.0073, 108.0076	1785	Cultivated
TắkRâng	27	TR23	144	Quảng Nam	Nam Trà My	Trà Cang	Tắk Râng	15.0073, 108.0076	1785	Cultivated
TắkRâng	32	TR28	145	Quảng Nam	Nam Trà My	Trà Cang	Tắk Râng	15.0073, 108.0076	1785	Cultivated
TắkRâng	26	TR22	146	Quảng Nam	Nam Trà My	Trà Cang	Tắk Râng	15.0073, 108.0076	1785	Cultivated
TắkRâng	15	TR11	147	Quảng Nam	Nam Trà My	Trà Cang	Tắk Râng	15.0073, 108.0076	1785	Cultivated
TắkRâng	13	TR9	148	Quảng Nam	Nam Trà My	Trà Cang	Tắk Râng	15.0073, 108.0076	1785	Cultivated
TắkRâng	12	TR8	149	Quảng Nam	Nam Trà My	Trà Cang	Tắk Râng	15.0073, 108.0076	1785	Cultivated
TắkRâng	9	TR5	150	Quảng Nam	Nam Trà My	Trà Cang	Tắk Râng	15.0073, 108.0076	1785	Cultivated
TắkRâng	11	TR7	152	Quảng Nam	Nam Trà My	Trà Cang	Tắk Râng	15.0073, 108.0076	1785	Cultivated
TắkRâng	16	TR12	153	Quảng Nam	Nam Trà My	Trà Cang	Tắk Râng	15.0073, 108.0076	1785	Cultivated
TắkRâng	23	TR19	203	Quảng Nam	Nam Trà My	Trà Cang	Tắk Râng	15.0073, 108.0076	1785	Cultivated
TắkRâng	21	TR17	204	Quảng Nam	Nam Trà My	Trà Cang	Tắk Râng	15.0073, 108.0076	1785	Cultivated
TắkRâng	20	TR16	205	Quảng Nam	Nam Trà My	Trà Cang	Tắk Râng	15.0073, 108.0076	1785	Cultivated
TắkRâng	17	TR13	206	Quảng Nam	Nam Trà My	Trà Cang	Tắk Râng	15.0073, 108.0076	1785	Cultivated
TắkRâng	22	TR18	207	Quảng Nam	Nam Trà My	Trà Cang	Tắk Râng	15.0073, 108.0076	1785	Cultivated
TắkRâng	19	TR15	208	Quảng Nam	Nam Trà My	Trà Cang	Tắk Râng	15.0073, 108.0076	1785	Cultivated
TắkRâng	24	TR20	209	Quảng Nam	Nam Trà My	Trà Cang	Tắk Râng	15.0073, 108.0076	1785	Cultivated
TắkRâng	18	TR14	210	Quảng Nam	Nam Trà My	Trà Cang	Tắk Râng	15.0073, 108.0076	1785	Cultivated
TắkRâng	7	TR3	275	Quảng Nam	Nam Trà My	Trà Cang	Tắk Râng	15.0073, 108.0076	1785	Cultivated
TắkRâng	33	TR29	318	Quảng Nam	Nam Trà My	Trà Cang	Tắk Râng	15.0073, 108.0076	1785	Cultivated
TắkRâng	35	TR31	320	Quảng Nam	Nam Trà My	Trà Cang	Tắk Râng	15.0073, 108.0076	1785	Cultivated
TắkRâng	34	TR30	322	Quảng Nam	Nam Trà My	Trà Cang	Tắk Râng	15.0073, 108.0076	1785	Cultivated
TắkNgo	32	TN32	76	Quảng Nam	Nam Trà My	Trà Linh	Tắk Ngo	15.0094, 108.0308	1650	Cultivated
TắkNgo	34	TN03-5	78	Quảng Nam	Nam Trà My	Trà Linh	Tắk Ngo	15.0094, 108.0308	1650	Cultivated
TắkNgo	33	TN02-5	80	Quảng Nam	Nam Trà My	Trà Linh	Tắk Ngo	15.0094, 108.0308	1650	Cultivated
TắkNgo	30	TN30	113	Quảng Nam	Nam Trà My	Trà Linh	Tắk Ngo	15.0094, 108.0308	1650	Cultivated
TắkNgo	28	TN28	114	Quảng Nam	Nam Trà My	Trà Linh	Tắk Ngo	15.0094, 108.0308	1650	Cultivated
TắkNgo	27	TN27	115	Quảng Nam	Nam Trà My	Trà Linh	Tắk Ngo	15.0094, 108.0308	1650	Cultivated
TắkNgo	24	TN24	116	Quảng Nam	Nam Trà My	Trà Linh	Tắk Ngo	15.0094, 108.0308	1650	Cultivated
TắkNgo	29	TN29	117	Quảng Nam	Nam Trà My	Trà Linh	Tắk Ngo	15.0094, 108.0308	1650	Cultivated
TắkNgo	26	TN26	118	Quảng Nam	Nam Trà My	Trà Linh	Tắk Ngo	15.0094, 108.0308	1650	Cultivated
TắkNgo	31	TN31	119	Quảng Nam	Nam Trà My	Trà Linh	Tắk Ngo	15.0094, 108.0308	1650	Cultivated

TắkNgo	25	TN25	120	Quảng Nam	Nam Trà My	Trà Linh	Tắk Ngo	15.0094, 108.0308	1650	Cultivated
TắkNgo	22	TN22	187	Quảng Nam	Nam Trà My	Trà Linh	Tắk Ngo	15.0094, 108.0308	1650	Cultivated
TắkNgo	20	TN20	188	Quảng Nam	Nam Trà My	Trà Linh	Tắk Ngo	15.0094, 108.0308	1650	Cultivated
TắkNgo	19	TN19	189	Quảng Nam	Nam Trà My	Trà Linh	Tắk Ngo	15.0094, 108.0308	1650	Cultivated
TắkNgo	16	TN16	190	Quảng Nam	Nam Trà My	Trà Linh	Tắk Ngo	15.0094, 108.0308	1650	Cultivated
TắkNgo	21	TN21	191	Quảng Nam	Nam Trà My	Trà Linh	Tắk Ngo	15.0094, 108.0308	1650	Cultivated
TắkNgo	18	TN18	192	Quảng Nam	Nam Trà My	Trà Linh	Tắk Ngo	15.0094, 108.0308	1650	Cultivated
TắkNgo	23	TN23	193	Quảng Nam	Nam Trà My	Trà Linh	Tắk Ngo	15.0094, 108.0308	1650	Cultivated
TắkNgo	17	TN17	194	Quảng Nam	Nam Trà My	Trà Linh	Tắk Ngo	15.0094, 108.0308	1650	Cultivated
TắkNgo	14	TN14	251	Quảng Nam	Nam Trà My	Trà Linh	Tắk Ngo	15.0094, 108.0308	1650	Cultivated
TắkNgo	12	TN12	252	Quảng Nam	Nam Trà My	Trà Linh	Tắk Ngo	15.0094, 108.0308	1650	Cultivated
TắkNgo	11	TN11	253	Quảng Nam	Nam Trà My	Trà Linh	Tắk Ngo	15.0094, 108.0308	1650	Cultivated
TắkNgo	8	TN8	254	Quảng Nam	Nam Trà My	Trà Linh	Tắk Ngo	15.0094, 108.0308	1650	Cultivated
TắkNgo	13	TN13	255	Quảng Nam	Nam Trà My	Trà Linh	Tắk Ngo	15.0094, 108.0308	1650	Cultivated
TắkNgo	10	TN10	256	Quảng Nam	Nam Trà My	Trà Linh	Tắk Ngo	15.0094, 108.0308	1650	Cultivated
TắkNgo	15	TN15	257	Quảng Nam	Nam Trà My	Trà Linh	Tắk Ngo	15.0094, 108.0308	1650	Cultivated
TắkNgo	9	TN9	258	Quảng Nam	Nam Trà My	Trà Linh	Tắk Ngo	15.0094, 108.0308	1650	Cultivated
TắkNgo	6	TN6	267	Quảng Nam	Nam Trà My	Trà Linh	Tắk Ngo	15.0094, 108.0308	1650	Cultivated
TắkNgo	4	TN4	268	Quảng Nam	Nam Trà My	Trà Linh	Tắk Ngo	15.0094, 108.0308	1650	Cultivated
TắkNgo	3	TN3	269	Quảng Nam	Nam Trà My	Trà Linh	Tắk Ngo	15.0094, 108.0308	1650	Cultivated
TắkNgo	5	TN5	271	Quảng Nam	Nam Trà My	Trà Linh	Tắk Ngo	15.0094, 108.0308	1650	Cultivated
TắkNgo	2	TN2	272	Quảng Nam	Nam Trà My	Trà Linh	Tắk Ngo	15.0094, 108.0308	1650	Cultivated
TắkNgo	7	TN7	273	Quảng Nam	Nam Trà My	Trà Linh	Tắk Ngo	15.0094, 108.0308	1650	Cultivated
TắkNgo	1	TN1	274	Quảng Nam	Nam Trà My	Trà Linh	Tắk Ngo	15.0094, 108.0308	1650	Cultivated
TắkLan	30	TL30	36	Quảng Nam	Nam Trà My	Trà Linh	Tắk Lan	15.0117, 108.0076	1915	Cultivated
TắkLan	12	TL12	49	Quảng Nam	Nam Trà My	Trà Linh	Tắk Lan	15.0117, 108.0076	1915	Cultivated
TắkLan	10	TL10	50	Quảng Nam	Nam Trà My	Trà Linh	Tắk Lan	15.0117, 108.0076	1915	Cultivated
TắkLan	9	TL9	51	Quảng Nam	Nam Trà My	Trà Linh	Tắk Lan	15.0117, 108.0076	1915	Cultivated
TắkLan	6	TL6	52	Quảng Nam	Nam Trà My	Trà Linh	Tắk Lan	15.0117, 108.0076	1915	Cultivated
TắkLan	11	TL11	53	Quảng Nam	Nam Trà My	Trà Linh	Tắk Lan	15.0117, 108.0076	1915	Cultivated
TắkLan	8	TL8	54	Quảng Nam	Nam Trà My	Trà Linh	Tắk Lan	15.0117, 108.0076	1915	Cultivated
TắkLan	13	TL13	55	Quảng Nam	Nam Trà My	Trà Linh	Tắk Lan	15.0117, 108.0076	1915	Cultivated
TắkLan	7	TL7	56	Quảng Nam	Nam Trà My	Trà Linh	Tắk Lan	15.0117, 108.0076	1915	Cultivated
TắkLan	4	TL4	73	Quảng Nam	Nam Trà My	Trà Linh	Tắk Lan	15.0117, 108.0076	1915	Cultivated
TắkLan	2	TL2	74	Quảng Nam	Nam Trà My	Trà Linh	Tắk Lan	15.0117, 108.0076	1915	Cultivated
TắkLan	1	TL1	75	Quảng Nam	Nam Trà My	Trà Linh	Tắk Lan	15.0117, 108.0076	1915	Cultivated
TắkLan	3	TL3	77	Quảng Nam	Nam Trà My	Trà Linh	Tắk Lan	15.0117, 108.0076	1915	Cultivated
TắkLan	5	TL5	79	Quảng Nam	Nam Trà My	Trà Linh	Tắk Lan	15.0117, 108.0076	1915	Cultivated

TắkLan	28	TL28	81	Quảng Nam	Nam Trà My	Trà Linh	Tắk Lan	15.0117, 108.0076	1915	Cultivated
TắkLan	26	TL26	82	Quảng Nam	Nam Trà My	Trà Linh	Tắk Lan	15.0117, 108.0076	1915	Cultivated
TắkLan	25	TL25	83	Quảng Nam	Nam Trà My	Trà Linh	Tắk Lan	15.0117, 108.0076	1915	Cultivated
TắkLan	22	TL22	84	Quảng Nam	Nam Trà My	Trà Linh	Tắk Lan	15.0117, 108.0076	1915	Cultivated
TắkLan	27	TL27	85	Quảng Nam	Nam Trà My	Trà Linh	Tắk Lan	15.0117, 108.0076	1915	Cultivated
TắkLan	24	TL24	86	Quảng Nam	Nam Trà My	Trà Linh	Tắk Lan	15.0117, 108.0076	1915	Cultivated
TắkLan	29	TL29	87	Quảng Nam	Nam Trà My	Trà Linh	Tắk Lan	15.0117, 108.0076	1915	Cultivated
TắkLan	23	TL23	88	Quảng Nam	Nam Trà My	Trà Linh	Tắk Lan	15.0117, 108.0076	1915	Cultivated
TắkLan	20	TL20	97	Quảng Nam	Nam Trà My	Trà Linh	Tắk Lan	15.0117, 108.0076	1915	Cultivated
TắkLan	18	TL18	98	Quảng Nam	Nam Trà My	Trà Linh	Tắk Lan	15.0117, 108.0076	1915	Cultivated
TắkLan	17	TL17	99	Quảng Nam	Nam Trà My	Trà Linh	Tắk Lan	15.0117, 108.0076	1915	Cultivated
TắkLan	14	TL14	100	Quảng Nam	Nam Trà My	Trà Linh	Tắk Lan	15.0117, 108.0076	1915	Cultivated
TắkLan	19	TL19	101	Quảng Nam	Nam Trà My	Trà Linh	Tắk Lan	15.0117, 108.0076	1915	Cultivated
TắkLan	16	TL16	102	Quảng Nam	Nam Trà My	Trà Linh	Tắk Lan	15.0117, 108.0076	1915	Cultivated
TắkLan	21	TL21	103	Quảng Nam	Nam Trà My	Trà Linh	Tắk Lan	15.0117, 108.0076	1915	Cultivated
TắkLan	15	TL15	104	Quảng Nam	Nam Trà My	Trà Linh	Tắk Lan	15.0117, 108.0076	1915	Cultivated
ConPin	16	CP16	156	Quảng Nam	Nam Trà My	Trà Linh	Con Pin	15.0140, 108.0310	1361	Cultivated
ConPin	18	CP18	155	Quảng Nam	Nam Trà My	Trà Linh	Con Pin	15.0140, 108.0310	1361	Cultivated
ConPin	15	CP15	157	Quảng Nam	Nam Trà My	Trà Linh	Con Pin	15.0140, 108.0310	1361	Cultivated
ConPin	12	CP12	158	Quảng Nam	Nam Trà My	Trà Linh	Con Pin	15.0140, 108.0310	1361	Cultivated
ConPin	17	CP17	159	Quảng Nam	Nam Trà My	Trà Linh	Con Pin	15.0140, 108.0310	1361	Cultivated
ConPin	14	CP14	160	Quảng Nam	Nam Trà My	Trà Linh	Con Pin	15.0140, 108.0310	1361	Cultivated
ConPin	19	CP19	161	Quảng Nam	Nam Trà My	Trà Linh	Con Pin	15.0140, 108.0310	1361	Cultivated
ConPin	13	CP13	162	Quảng Nam	Nam Trà My	Trà Linh	Con Pin	15.0140, 108.0310	1361	Cultivated
ConPin	10	CP10	195	Quảng Nam	Nam Trà My	Trà Linh	Con Pin	15.0140, 108.0310	1361	Cultivated
ConPin	8	CP8	196	Quảng Nam	Nam Trà My	Trà Linh	Con Pin	15.0140, 108.0310	1361	Cultivated
ConPin	7	CP7	197	Quảng Nam	Nam Trà My	Trà Linh	Con Pin	15.0140, 108.0310	1361	Cultivated
ConPin	4	CP4	198	Quảng Nam	Nam Trà My	Trà Linh	Con Pin	15.0140, 108.0310	1361	Cultivated
ConPin	9	CP9	199	Quảng Nam	Nam Trà My	Trà Linh	Con Pin	15.0140, 108.0310	1361	Cultivated
ConPin	6	CP6	200	Quảng Nam	Nam Trà My	Trà Linh	Con Pin	15.0140, 108.0310	1361	Cultivated
ConPin	11	CP11	201	Quảng Nam	Nam Trà My	Trà Linh	Con Pin	15.0140, 108.0310	1361	Cultivated
ConPin	5	CP5	202	Quảng Nam	Nam Trà My	Trà Linh	Con Pin	15.0140, 108.0310	1361	Cultivated
ConPin	2	CP2	243	Quảng Nam	Nam Trà My	Trà Linh	Con Pin	15.0140, 108.0310	1361	Cultivated
ConPin	1	CP1	247	Quảng Nam	Nam Trà My	Trà Linh	Con Pin	15.0140, 108.0310	1361	Cultivated
ConPin	3	CP3	249	Quảng Nam	Nam Trà My	Trà Linh	Con Pin	15.0140, 108.0310	1361	Cultivated
ConPin	20	CP20	270	Quảng Nam	Nam Trà My	Trà Linh	Con Pin	15.0140, 108.0310	1361	Cultivated
TràLinh_drug_materials_farm	7	Trà Linh 7	39	Quảng Nam	Nam Trà My	Trà Linh	Trà Linh drug materials farm	15.0318, 107.9791	1835	Cultivated
TràLinh_drug_materials_farm	30	Trà Linh 30	9	Quảng Nam	Nam Trà My	Trà Linh	Trà Linh drug materials farm	15.0318, 107.9791	1835	Cultivated

TràLinh_drug_materials_farm	28	Trà Linh 28-duplicate	10	Quảng Nam	Nam Trà My	Trà Linh	Trà Linh drug materials farm	15.0318, 107.9791	1835	Cultivated
TràLinh_drug_materials_farm	27	Trà Linh 27	11	Quảng Nam	Nam Trà My	Trà Linh	Trà Linh drug materials farm	15.0318, 107.9791	1835	Cultivated
TràLinh_drug_materials_farm	24	Trà Linh 24	12	Quảng Nam	Nam Trà My	Trà Linh	Trà Linh drug materials farm	15.0318, 107.9791	1835	Cultivated
TràLinh_drug_materials_farm	29	Trà Linh 29	13	Quảng Nam	Nam Trà My	Trà Linh	Trà Linh drug materials farm	15.0318, 107.9791	1835	Cultivated
TràLinh_drug_materials_farm	26	Trà Linh 26	14	Quảng Nam	Nam Trà My	Trà Linh	Trà Linh drug materials farm	15.0318, 107.9791	1835	Cultivated
TràLinh_drug_materials_farm	31	Trà Linh 31	15	Quảng Nam	Nam Trà My	Trà Linh	Trà Linh drug materials farm	15.0318, 107.9791	1835	Cultivated
TràLinh_drug_materials_farm	25	Trà Linh 25	16	Quảng Nam	Nam Trà My	Trà Linh	Trà Linh drug materials farm	15.0318, 107.9791	1835	Cultivated
TràLinh_drug_materials_farm	6	Trà Linh 6	33	Quảng Nam	Nam Trà My	Trà Linh	Trà Linh drug materials farm	15.0318, 107.9791	1835	Cultivated
TràLinh_drug_materials_farm	4	Trà Linh 4	34	Quảng Nam	Nam Trà My	Trà Linh	Trà Linh drug materials farm	15.0318, 107.9791	1835	Cultivated
TràLinh_drug_materials_farm	3	Trà Linh 3	35	Quảng Nam	Nam Trà My	Trà Linh	Trà Linh drug materials farm	15.0318, 107.9791	1835	Cultivated
TràLinh_drug_materials_farm	5	Trà Linh 5	37	Quảng Nam	Nam Trà My	Trà Linh	Trà Linh drug materials farm	15.0318, 107.9791	1835	Cultivated
TràLinh_drug_materials_farm	2	Trà Linh 2	38	Quảng Nam	Nam Trà My	Trà Linh	Trà Linh drug materials farm	15.0318, 107.9791	1835	Cultivated
TràLinh_drug_materials_farm	1	Trà Linh 1	40	Quảng Nam	Nam Trà My	Trà Linh	Trà Linh drug materials farm	15.0318, 107.9791	1835	Cultivated
TràLinh_drug_materials_farm	14	Trà Linh 14	65	Quảng Nam	Nam Trà My	Trà Linh	Trà Linh drug materials farm	15.0318, 107.9791	1835	Cultivated
TràLinh_drug_materials_farm	12	Trà Linh 12	66	Quảng Nam	Nam Trà My	Trà Linh	Trà Linh drug materials farm	15.0318, 107.9791	1835	Cultivated
TràLinh_drug_materials_farm	11	Trà Linh 11	67	Quảng Nam	Nam Trà My	Trà Linh	Trà Linh drug materials farm	15.0318, 107.9791	1835	Cultivated
TràLinh_drug_materials_farm	8	Trà Linh 8	68	Quảng Nam	Nam Trà My	Trà Linh	Trà Linh drug materials farm	15.0318, 107.9791	1835	Cultivated
TràLinh_drug_materials_farm	13	Trà Linh 13	69	Quảng Nam	Nam Trà My	Trà Linh	Trà Linh drug materials farm	15.0318, 107.9791	1835	Cultivated
TràLinh_drug_materials_farm	10	Trà Linh 10	70	Quảng Nam	Nam Trà My	Trà Linh	Trà Linh drug materials farm	15.0318, 107.9791	1835	Cultivated
TràLinh_drug_materials_farm	15	Trà Linh 15	71	Quảng Nam	Nam Trà My	Trà Linh	Trà Linh drug materials farm	15.0318, 107.9791	1835	Cultivated
TràLinh_drug_materials_farm	9	Trà Linh 9	72	Quảng Nam	Nam Trà My	Trà Linh	Trà Linh drug materials farm	15.0318, 107.9791	1835	Cultivated
TràLinh_drug_materials_farm	22	Trà Linh 22	89	Quảng Nam	Nam Trà My	Trà Linh	Trà Linh drug materials farm	15.0318, 107.9791	1835	Cultivated
TràLinh_drug_materials_farm	20	Trà Linh 20	90	Quảng Nam	Nam Trà My	Trà Linh	Trà Linh drug materials farm	15.0318, 107.9791	1835	Cultivated
TràLinh_drug_materials_farm	19	Trà Linh 19	91	Quảng Nam	Nam Trà My	Trà Linh	Trà Linh drug materials farm	15.0318, 107.9791	1835	Cultivated
TràLinh_drug_materials_farm	16	Trà Linh 16	92	Quảng Nam	Nam Trà My	Trà Linh	Trà Linh drug materials farm	15.0318, 107.9791	1835	Cultivated
TràLinh_drug_materials_farm	21	Trà Linh 21	93	Quảng Nam	Nam Trà My	Trà Linh	Trà Linh drug materials farm	15.0318, 107.9791	1835	Cultivated
TràLinh_drug_materials_farm	18	Trà Linh 18	94	Quảng Nam	Nam Trà My	Trà Linh	Trà Linh drug materials farm	15.0318, 107.9791	1835	Cultivated
TràLinh_drug_materials_farm	23	Trà Linh 23	95	Quảng Nam	Nam Trà My	Trà Linh	Trà Linh drug materials farm	15.0318, 107.9791	1835	Cultivated
TràLinh_drug_materials_farm	17	Trà Linh 17	96	Quảng Nam	Nam Trà My	Trà Linh	Trà Linh drug materials farm	15.0318, 107.9791	1835	Cultivated
MăngLùng	12	ML03-3	163	Quảng Nam	Nam Trà My	Trà Linh	Măng Lùng	15.0320, 107.9790	1846	Cultivated
MăngLùng	10	ML03-1	164	Quảng Nam	Nam Trà My	Trà Linh	Măng Lùng	15.0320, 107.9790	1846	Cultivated
MăngLùng	9	ML02-4	165	Quảng Nam	Nam Trà My	Trà Linh	Măng Lùng	15.0320, 107.9790	1846	Cultivated
MăngLùng	6	ML02-1	166	Quảng Nam	Nam Trà My	Trà Linh	Măng Lùng	15.0320, 107.9790	1846	Cultivated
MăngLùng	11	ML03-2	167	Quảng Nam	Nam Trà My	Trà Linh	Măng Lùng	15.0320, 107.9790	1846	Cultivated
MăngLùng	8	ML02-3	168	Quảng Nam	Nam Trà My	Trà Linh	Măng Lùng	15.0320, 107.9790	1846	Cultivated
MăngLùng	13	ML03-4	169	Quảng Nam	Nam Trà My	Trà Linh	Măng Lùng	15.0320, 107.9790	1846	Cultivated
MăngLùng	7	ML02-2	170	Quảng Nam	Nam Trà My	Trà Linh	Măng Lùng	15.0320, 107.9790	1846	Cultivated
MăngLùng	26	ML07-2	217	Quảng Nam	Nam Trà My	Trà Linh	Măng Lùng	15.0320, 107.9790	1846	Cultivated

MăngLùng	29	ML08-1	219	Quảng Nam	Nam Trà My	Trà Linh	Măng Lùng	15.0320, 107.9790	1846	Cultivated
MăngLùng	27	ML07-3	220	Quảng Nam	Nam Trà My	Trà Linh	Măng Lùng	15.0320, 107.9790	1846	Cultivated
MăngLùng	25	ML07-1	221	Quảng Nam	Nam Trà My	Trà Linh	Măng Lùng	15.0320, 107.9790	1846	Cultivated
MăngLùng	22	ML06-2	222	Quảng Nam	Nam Trà My	Trà Linh	Măng Lùng	15.0320, 107.9790	1846	Cultivated
MăngLùng	28	ML07-4	223	Quảng Nam	Nam Trà My	Trà Linh	Măng Lùng	15.0320, 107.9790	1846	Cultivated
MăngLùng	24	ML06-4	224	Quảng Nam	Nam Trà My	Trà Linh	Măng Lùng	15.0320, 107.9790	1846	Cultivated
MăngLùng	30	ML08-2	225	Quảng Nam	Nam Trà My	Trà Linh	Măng Lùng	15.0320, 107.9790	1846	Cultivated
MăngLùng	23	ML06-3	226	Quảng Nam	Nam Trà My	Trà Linh	Măng Lùng	15.0320, 107.9790	1846	Cultivated
MăngLùng	31	ML08-3	242	Quảng Nam	Nam Trà My	Trà Linh	Măng Lùng	15.0320, 107.9790	1846	Cultivated
MăngLùng	4	ML01-4	315	Quảng Nam	Nam Trà My	Trà Linh	Măng Lùng	15.0320, 107.9790	1846	Cultivated
MăngLùng	2	ML01-2	316	Quảng Nam	Nam Trà My	Trà Linh	Măng Lùng	15.0320, 107.9790	1846	Cultivated
MăngLùng	1	ML01-1	317	Quảng Nam	Nam Trà My	Trà Linh	Măng Lùng	15.0320, 107.9790	1846	Cultivated
MăngLùng	3	ML01-3	319	Quảng Nam	Nam Trà My	Trà Linh	Măng Lùng	15.0320, 107.9790	1846	Cultivated
MăngLùng	5	ML01-5	321	Quảng Nam	Nam Trà My	Trà Linh	Măng Lùng	15.0320, 107.9790	1846	Cultivated
MăngLùng	20	ML05-3	323	Quảng Nam	Nam Trà My	Trà Linh	Măng Lùng	15.0320, 107.9790	1846	Cultivated
MăngLùng	18	ML05-1	324	Quảng Nam	Nam Trà My	Trà Linh	Măng Lùng	15.0320, 107.9790	1846	Cultivated
MăngLùng	17	ML04-4	325	Quảng Nam	Nam Trà My	Trà Linh	Măng Lùng	15.0320, 107.9790	1846	Cultivated
MăngLùng	14	ML04-1	326	Quảng Nam	Nam Trà My	Trà Linh	Măng Lùng	15.0320, 107.9790	1846	Cultivated
MăngLùng	19	ML05-2	327	Quảng Nam	Nam Trà My	Trà Linh	Măng Lùng	15.0320, 107.9790	1846	Cultivated
MăngLùng	16	ML04-3	328	Quảng Nam	Nam Trà My	Trà Linh	Măng Lùng	15.0320, 107.9790	1846	Cultivated
MăngLùng	21	ML05-4	329	Quảng Nam	Nam Trà My	Trà Linh	Măng Lùng	15.0320, 107.9790	1846	Cultivated
MăngLùng	15	ML04-2	330	Quảng Nam	Nam Trà My	Trà Linh	Măng Lùng	15.0320, 107.9790	1846	Cultivated
MăngLùng	46	ML15	171	Quảng Nam	Nam Trà My	Trà Linh	Măng Lùng	15.0321, 107.9843	1820	Cultivated
MăngLùng	44	ML13	172	Quảng Nam	Nam Trà My	Trà Linh	Măng Lùng	15.0321, 107.9843	1820	Cultivated
MăngLùng	43	ML12	173	Quảng Nam	Nam Trà My	Trà Linh	Măng Lùng	15.0321, 107.9843	1820	Cultivated
MăngLùng	40	ML9	174	Quảng Nam	Nam Trà My	Trà Linh	Măng Lùng	15.0321, 107.9843	1820	Cultivated
MăngLùng	45	ML14	175	Quảng Nam	Nam Trà My	Trà Linh	Măng Lùng	15.0321, 107.9843	1820	Cultivated
MăngLùng	42	ML11	176	Quảng Nam	Nam Trà My	Trà Linh	Măng Lùng	15.0321, 107.9843	1820	Cultivated
MăngLùng	47	ML16	177	Quảng Nam	Nam Trà My	Trà Linh	Măng Lùng	15.0321, 107.9843	1820	Cultivated
MăngLùng	41	ML10	178	Quảng Nam	Nam Trà My	Trà Linh	Măng Lùng	15.0321, 107.9843	1820	Cultivated
MăngLùng	60	ML29	244	Quảng Nam	Nam Trà My	Trà Linh	Măng Lùng	15.0321, 107.9843	1820	Cultivated
MăngLùng	59	ML28	245	Quảng Nam	Nam Trà My	Trà Linh	Măng Lùng	15.0321, 107.9843	1820	Cultivated
MăngLùng	56	ML25	246	Quảng Nam	Nam Trà My	Trà Linh	Măng Lùng	15.0321, 107.9843	1820	Cultivated
MăngLùng	58	ML27	248	Quảng Nam	Nam Trà My	Trà Linh	Măng Lùng	15.0321, 107.9843	1820	Cultivated
MăngLùng	57	ML26	250	Quảng Nam	Nam Trà My	Trà Linh	Măng Lùng	15.0321, 107.9843	1820	Cultivated
MăngLùng	38	ML7	291	Quảng Nam	Nam Trà My	Trà Linh	Măng Lùng	15.0321, 107.9843	1820	Cultivated
MăngLùng	36	ML5	292	Quảng Nam	Nam Trà My	Trà Linh	Măng Lùng	15.0321, 107.9843	1820	Cultivated
MăngLùng	35	ML4	293	Quảng Nam	Nam Trà My	Trà Linh	Măng Lùng	15.0321, 107.9843	1820	Cultivated

MăngLùng	32	ML1	294	Quảng Nam	Nam Trà My	Trà Linh	Măng Lùng	15.0321, 107.9843	1820	Cultivated
MăngLùng	37	ML6	295	Quảng Nam	Nam Trà My	Trà Linh	Măng Lùng	15.0321, 107.9843	1820	Cultivated
MăngLùng	34	ML3	296	Quảng Nam	Nam Trà My	Trà Linh	Măng Lùng	15.0321, 107.9843	1820	Cultivated
MăngLùng	39	ML8	297	Quảng Nam	Nam Trà My	Trà Linh	Măng Lùng	15.0321, 107.9843	1820	Cultivated
MăngLùng	33	ML2	298	Quảng Nam	Nam Trà My	Trà Linh	Măng Lùng	15.0321, 107.9843	1820	Cultivated
MăngLùng	54	ML23	307	Quảng Nam	Nam Trà My	Trà Linh	Măng Lùng	15.0321, 107.9843	1820	Cultivated
MăngLùng	52	ML21	308	Quảng Nam	Nam Trà My	Trà Linh	Măng Lùng	15.0321, 107.9843	1820	Cultivated
MăngLùng	51	ML20	309	Quảng Nam	Nam Trà My	Trà Linh	Măng Lùng	15.0321, 107.9843	1820	Cultivated
MăngLùng	48	ML17	310	Quảng Nam	Nam Trà My	Trà Linh	Măng Lùng	15.0321, 107.9843	1820	Cultivated
MăngLùng	53	ML22	311	Quảng Nam	Nam Trà My	Trà Linh	Măng Lùng	15.0321, 107.9843	1820	Cultivated
MăngLùng	50	ML19	312	Quảng Nam	Nam Trà My	Trà Linh	Măng Lùng	15.0321, 107.9843	1820	Cultivated
MăngLùng	55	ML24	313	Quảng Nam	Nam Trà My	Trà Linh	Măng Lùng	15.0321, 107.9843	1820	Cultivated
MăngLùng	49	ML18	314	Quảng Nam	Nam Trà My	Trà Linh	Măng Lùng	15.0321, 107.9843	1820	Cultivated
TràLinh_H3	3	TL02-2	46	Quảng Nam	Nam Trà My	Trà Linh	Hamlet 3	15.0352, 107.9887	1473	Cultivated
TràLinh_H3	9	TL05-3	4	Quảng Nam	Nam Trà My	Trà Linh	Hamlet 3	15.0352, 107.9887	1473	Cultivated
TràLinh_H3	11	TL08-2	6	Quảng Nam	Nam Trà My	Trà Linh	Hamlet 3	15.0352, 107.9887	1473	Cultivated
TràLinh_H3	10	TL07-4	8	Quảng Nam	Nam Trà My	Trà Linh	Hamlet 3	15.0352, 107.9887	1473	Cultivated
TràLinh_H3	7	TL04-2	41	Quảng Nam	Nam Trà My	Trà Linh	Hamlet 3	15.0352, 107.9887	1473	Cultivated
TràLinh_H3	5	TL02-5	42	Quảng Nam	Nam Trà My	Trà Linh	Hamlet 3	15.0352, 107.9887	1473	Cultivated
TràLinh_H3	4	TL02-3	43	Quảng Nam	Nam Trà My	Trà Linh	Hamlet 3	15.0352, 107.9887	1473	Cultivated
TràLinh_H3	1	TL01-3	44	Quảng Nam	Nam Trà My	Trà Linh	Hamlet 3	15.0352, 107.9887	1473	Cultivated
TràLinh_H3	6	TL03-4	45	Quảng Nam	Nam Trà My	Trà Linh	Hamlet 3	15.0352, 107.9887	1473	Cultivated
TràLinh_H3	8	TL05-2	47	Quảng Nam	Nam Trà My	Trà Linh	Hamlet 3	15.0352, 107.9887	1473	Cultivated
TràLinh_H3	2	TL01-5	48	Quảng Nam	Nam Trà My	Trà Linh	Hamlet 3	15.0352, 107.9887	1473	Cultivated
MôLút	1	NL1	111	Kon Tum	Đắk Glei	Ngọc Linh	Mô Lút	15.0620, 107.9440	1768	Wild
Xốp	1	X1	105	Kon Tum	Đắk Glei	Xốp		15.0760, 107.8320	1299	Wild
TràCang	2	Trà Cang 2	280	Quảng Nam	Nam Trà My	Trà Cang	Hamlet 3	15.0854, 108.0485	1010	Cultivated
TràCang	1	Trà Cang 1	305	Quảng Nam	Nam Trà My	Trà Cang	Hamlet 3	15.0854, 108.0485	1010	Cultivated
TràCang	4	Trà Cang 4	277	Quảng Nam	Nam Trà My	Trà Cang	Hamlet 2	15.1036, 108.0615	1029	Cultivated
TràCang	3	Trà Cang 3	238	Quảng Nam	Nam Trà My	Trà Cang	Hamlet 2	15.1036, 108.0615	1029	Cultivated
MườngHoong	4	MH4	109	Kon Tum	Đắk Glei	Mường Hoong		15.1250, 107.9119	1395	Wild
MườngHoong	3	MH3	106	Kon Tum	Đắk Glei	Mường Hoong		15.1250, 107.9120	1395	Wild
MườngHoong	2	MH2	107	Kon Tum	Đắk Glei	Mường Hoong		15.1250, 107.9121	1395	Wild
MườngHoong	1	MH1	110	Kon Tum	Đắk Glei	Mường Hoong		15.1250, 107.9122	1395	Wild
PhướcLộc	11	PL06-4	288	Quảng Nam	Phước Sơn	Phước Lộc		15.2750, 107.8790	687	Wild
PhướcLộc	8	PL05-2	211	Quảng Nam	Phước Sơn	Phước Lộc		15.2750, 107.8790	687	Wild
PhướcLộc	6	PL04-5	212	Quảng Nam	Phước Sơn	Phước Lộc		15.2750, 107.8790	687	Wild
PhướcLộc	5	PL04-4	213	Quảng Nam	Phước Sơn	Phước Lộc		15.2750, 107.8790	687	Wild

PhướcLộc	2	PL04-1	214	Quảng Nam	Phước Sơn	Phước Lộc		15.2750, 107.8790	687	Wild
PhướcLộc	7	PL05-1	215	Quảng Nam	Phước Sơn	Phước Lộc		15.2750, 107.8790	687	Wild
PhướcLộc	4	PL04-3	216	Quảng Nam	Phước Sơn	Phước Lộc		15.2750, 107.8790	687	Wild
PhướcLộc	3	PL04-2	218	Quảng Nam	Phước Sơn	Phước Lộc		15.2750, 107.8790	687	Wild
PhướcLộc	1	PL02-3	241	Quảng Nam	Phước Sơn	Phước Lộc		15.2750, 107.8790	687	Wild
PhướcLộc	14	PL07-3	284	Quảng Nam	Phước Sơn	Phước Lộc		15.2750, 107.8790	687	Wild
PhướcLộc	12	PL06-5	285	Quảng Nam	Phước Sơn	Phước Lộc		15.2750, 107.8790	687	Wild
PhướcLộc	9	PL05-5	286	Quảng Nam	Phước Sơn	Phước Lộc		15.2750, 107.8790	687	Wild
PhướcLộc	10	PL06-3	290	Quảng Nam	Phước Sơn	Phước Lộc		15.2750, 107.8790	687	Wild
PhướcLộc	13	PL07-1	304	Quảng Nam	Phước Sơn	Phước Lộc		15.2750, 107.8790	687	Wild
Ch'Ơm	1	TG02	237	Quảng Nam	Tây Giang	Ch' Ơm		15.7920, 107.2590	1350	Cultivated
Ch'Ơm	3	TG04	239	Quảng Nam	Tây Giang	Ch' Ơm		15.7920, 107.2590	1350	Cultivated
Ch'Ơm	5	TG07	306	Quảng Nam	Tây Giang	Ch' Ơm		15.7920, 107.2590	1350	Cultivated
Ch'Ơm	4	TG05	235	Quảng Nam	Tây Giang	Ch' Ơm		15.7920, 107.2590	1350	Cultivated
Ch'Ơm	2	TG03	236	Quảng Nam	Tây Giang	Ch' Ơm		15.7920, 107.2590	1350	Cultivated
Ch'Ơm	6	TG06-1	302	Quảng Nam	Tây Giang	Ch' Ơm		15.7920, 107.2590	1350	Cultivated
