# Supplementary material for: Target Capture Reveals the Complex Origin of Vietnamese Ginseng
Source: Front Plant Sci. 2022 Jul 13;13:814178. doi: 10.3389/fpls.2022.814178 (PMC9326450; doi:10.3389/fpls.2022.814178)
Supplement: Supplementary Table S2 — Populations' information. [file Data_Sheet_2.PDF]

No.	Location				Population name	Coordinates	Cultivating status	Number of samples	Total
	Province	District	Commune	Locality					
1	Quảng Nam	Tây Giang	Ch' Ơm		Ch'Ơm	15.7920 N, 107.2590 E	Cultivated	6	319
2		Phước Sơn	Phước Lộc		PhướcLộc	15.2750 N, 107.8790 E	Wild	14	
3		Nam Trà My	Trà Nam		TràNam	15.0070 N, 108.0860 E	Cultivated	3	
4				Tắk Túi	TắkTúi	14.9641 N 108.1005 E	Cultivated	30	
5			Trà Cang	Hamlet 3	TràCang	15.0854 N, 108.0485 E	Cultivated	2	
				Hamlet 2		15.1036 N, 108.0615 E	Cultivated	2	
6				Tắk Râng	TắkRâng	15.0073 N, 108.0076 E	Cultivated	31	
7			Trà Linh	Măng Lùng	MăngLùng	15.0320 N, 107.9790 E	Cultivated	31	
						15.0321 N, 107.9843 E	Cultivated	29	
8				Con Pin	ConPin	15.0140 N, 108.0310 E	Cultivated	20	
9				Tắk Ngo	TắkNgo	15.0094 N, 108.0308 E	Cultivated	34	
10				Tắk Lan	TắkLan	15.0117 N, 108.0076 E	Cultivated	30	
11				Trà Linh Medicinal Plant Center	TLCenter	15.0318 N, 107.9791 E	Cultivated	31	
12				Hamlet 3	TràLinh_H3	15.0352 N, 107.9887 E	Cultivated	11	
13	Kon Tum	Tu Mơ Rông	Măng Ri	Chung Tam	ChungTam	14.9710 N, 107.8950 E	Cultivated	14	
14			Tê Xăng	Đắk Viên	ĐắkViên	14.9610 N, 107.9540 E	Cultivated	13	
15			Ngọk Lây	Lộc Bông	LộcBông	14.9850 N, 108.0300 E	Wild	7	
16				Măng Rương	MăngRương	14.9708 N, 107.9990 E	Cultivated	5	
17		Đắk Glei	Xốp		Xốp	15.0760 N, 107.8320 E	Wild	1	
18			Mường Hoong		MườngHoong	15.1250 N, 107.9119 E	Wild	4	
19			Ngọc Linh	Mô Lút 1	MôLút	15.0620 N, 107.9440 E	Wild	1	
