# Supplementary material for: Target Capture Reveals the Complex Origin of Vietnamese Ginseng
Source: Front Plant Sci. 2022 Jul 13;13:814178. doi: 10.3389/fpls.2022.814178 (PMC9326450; doi:10.3389/fpls.2022.814178)
Supplement: Supplementary file 13 [file Presentation_1.PDF]

Randomization SNPs

Assessment of the effect of linked SNPs on STRUCTURE analyses

```
knitr::opts_chunk$set(echo = TRUE)
```

Motivation

Despite all the advantages of target capture, this method can generate linked SNPs. The STRUCTURE program permits inclusion of weakly linked markers with some degree of non-independence. To overcome the possible effects of linked SNPs, we sub-sampled our data set. From the original data set, we created 10 sets where we randomly choose 1 SNP per marker. To analyse these 10 data sets, we used the same pipeline as for the original data set, using STRUCTURE with 3 replicates and for k=1 to k=8 for 100 000 generations. Then we compiled results for the 10 data sets to estimate the best number of ancestral populations and performed a t-test to evaluate if the sub sampled admixture values were significantly different from those obtained for the full SNPs data set.

Build the randomized sets

```
# Randomise 1 SNP per maker

mkdir -p ./markers
cat deepvariant.cohort_filtered_1181.vcf | grep "^#" > header.txt
cat deepvariant.cohort_filtered_1181.vcf | grep -v "^#" | cut -f1 |
sort  | uniq > ./markers/markers
for i in $(cat ./markers/markers); \
    do cat deepvariant.cohort_filtered_1181.vcf  | grep "^$i" > ./markers/"$i"_marker; \
done

ls ./markers/*_marker > list_file

mkdir -p ./randomized_vcfs

count=10
for J in $(seq $count);
    do for i in $(cat list_file); do shuf -n 1 $i >> ./randomized_vcfs/set_"$J".temp; \
    cat header.txt ./randomized_vcfs/set_"$J".temp > ./randomized_vcfs/set_"$J".vcf; \
    done;
    done
rm ./randomized_vcfs/*.temp
```

STRUCTURE run of the 10 randomized sets

```
#!/bin/bash

MAIN_DIR=/home/vincent/Project/Ginseng/4_Structure/0_analyses/2022-03/randomized_vcf_pgdspider
```

```
MAINP=/home/vincent/Project/Ginseng/4_Structure/0_analyses/2022-03/randomized_vcf_pgdspider/mainparams
EXP=/home/vincent/Project/Ginseng/4_Structure/0_analyses/2022-03/randomized_vcf_pgdspider/extraparams
cd randomized_vcf_pgdspider
for REP in $(seq 1 3); \
    do
        for INPUT in $(ls *vcf); \
            do
                for i in {1..10} #change number if necessary
                do
                    (cd $MAIN_DIR
                    mkdir -p "$INPUT"_"$i"."$REP"
                    cd "$INPUT"_"$i"."$REP"
                    pwd
                    structure -i ../"$INPUT" \
                    -o "$INPUT"_"$i"."$REP" \
                    -m $MAINP \
                    -e $EXP \
                    -K $i > run_"$INPUT"_"$i"."$REP".log
                    ) &
                done;
        done;
```

The rawsummary.tsv contain the likelihood scores of the 300 analyses. The ln score is used to decide which value K is more appropriate for our data set.

Randomized sets analysed individualy

```
knitr::opts_chunk$set(echo = TRUE)
library(tidyverse)
```

```
## -- Attaching packages --------------------------------------- tidyverse 1.3.1 --

## v ggplot2 3.3.6     v purrr   0.3.4
## v tibble  3.1.7     v dplyr   1.0.9
## v tidyr   1.2.0     v stringr 1.4.0
## v readr   2.1.2     v forcats 0.5.1

## -- Conflicts ------------------------------------------ tidyverse_conflicts() --
## x dplyr::filter() masks stats::filter()
## x dplyr::lag()    masks stats::lag()
```

```
library(viridis)
```

```
## Loading required package: viridisLite
```

```
library(hrbrthemes)
```

```
## NOTE: Either Arial Narrow or Roboto Condensed fonts are required to use these themes.

##       Please use hrbrthemes::import_roboto_condensed() to install Roboto Condensed and

##       if Arial Narrow is not on your system, please see https://bit.ly/arialnarrow
```

```
library(kableExtra)
```

```
##
## Attaching package: 'kableExtra'
```

```
## The following object is masked from 'package:dplyr':
##
##     group_rows
```

```
library(gridExtra)
```

```
##
## Attaching package: 'gridExtra'

## The following object is masked from 'package:dplyr':
##
##     combine
```

```
library(ggpubr)
library(rstatix)
```

```
##
## Attaching package: 'rstatix'

## The following object is masked from 'package:stats':
##
##     filter
```

```
setwd("~/Project/Ginseng/4_Structure/0_analyses/2022-03/randomized_vcf_pgdspider/harvester/")
```

Prepare the data

```
csv <- read.csv("rawsummary.tsv", sep = "\t")
mean_ln <- csv %>% group_by(set, k) %>%
  mutate(ln.mean = mean(.data$Est..Ln.prob..of.data[.data$REP])) %>%
  arrange(set, k)
sd_ln <- mean_ln %>% mutate(sd = Est..Ln.prob..of.data - ln.mean)
sd_ln_sd <- sd_ln %>% group_by(set, k) %>%
  mutate(ln_sd = sd(.data$sd[.data$REP])) %>%
  select(set, ln.mean, ln_sd) %>%
  unique() %>%
  filter_all(all_vars(set != "ALL SNPs")) %>%
  arrange(as.numeric(set)) %>%
  mutate(Set = recode(set, "1" = "Set 1", "2" = "Set 2", "3" = "Set 3",
                      "4" = "Set 4", "5" = "Set 5", "6" = "Set 6",
                      "7" = "Set 7", "8" = "Set 8", "9" = "Set 9",
                      "10" = "Set 10"))
```

```
## Adding missing grouping variables: `k`
```

L(K)(mean +- SD)

```
p <- sd_ln_sd %>%
  ggplot(aes(x = k, y = (ln.mean), color = factor(set))) +
  geom_point(size = 3) +
  geom_line() +
  scale_color_manual("Randomized sets", values=c("#9e0142", "#d53e4f", "#f46d43",
                                                 "#fdae61", "#fee08b", "#e6f598",
                                                 "#abdda4", "#66c2a5", "#3288bd",
                                                 "#5e4fa2"), breaks =c(1,2,3,4,5,6,7,8,9,10)) +
  theme_classic() +
```

```
  theme(plot.title = element_text(hjust = 1)) +
  xlab("K") + ylab("Mean of est. Ln prob of data") +
  geom_errorbar(aes(ymin = ln.mean - ln_sd, ymax = ln.mean + ln_sd), width = 0.15)+
  ggtitle("L(K)(mean +-SD") +
  xlim("1","2","3","4", "5", "6", "7", "8", "9", "10")
p
```

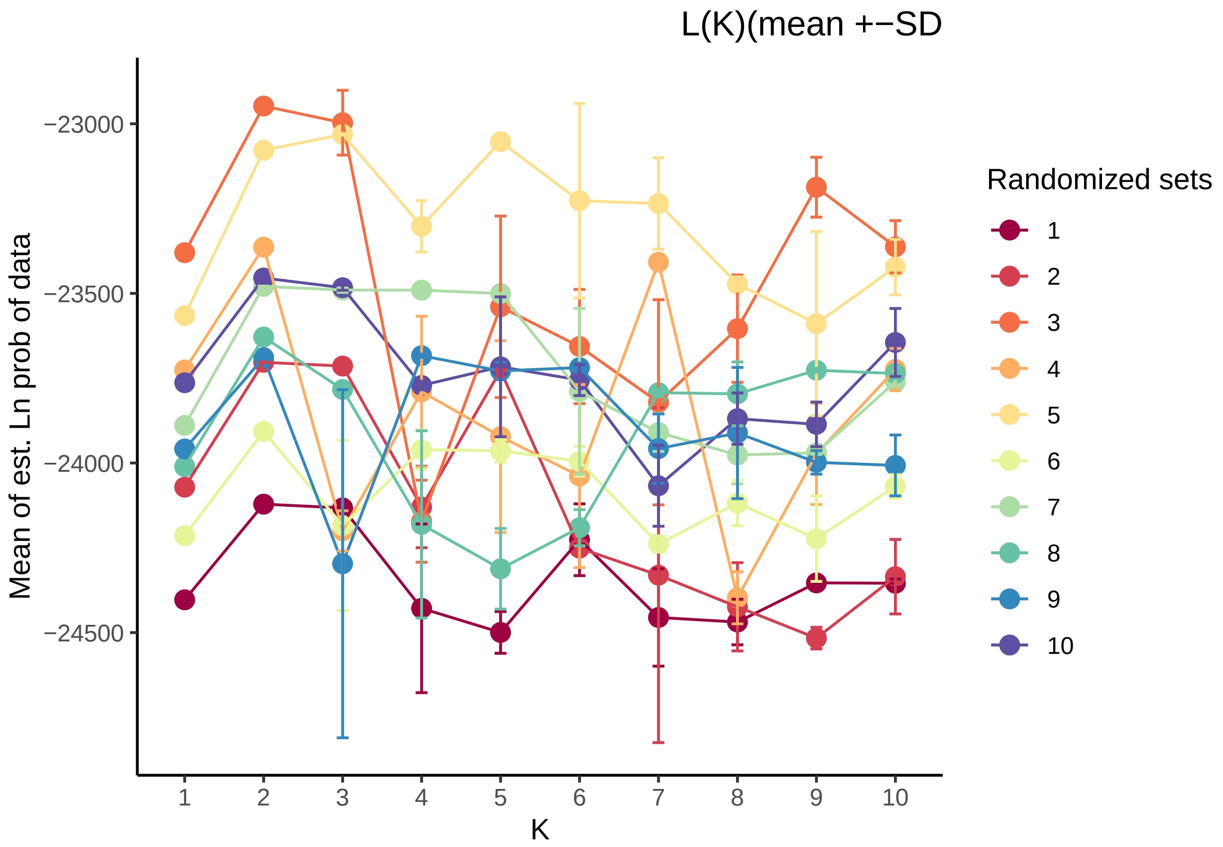


```
p + facet_wrap(~factor(Set, levels = c("Set 1", "Set 2", "Set 3", "Set 4", "
                                          Set 5", "Set 6", "Set 7", "Set 8",
                                          "Set 9", "Set 10")), scale = "free")
```

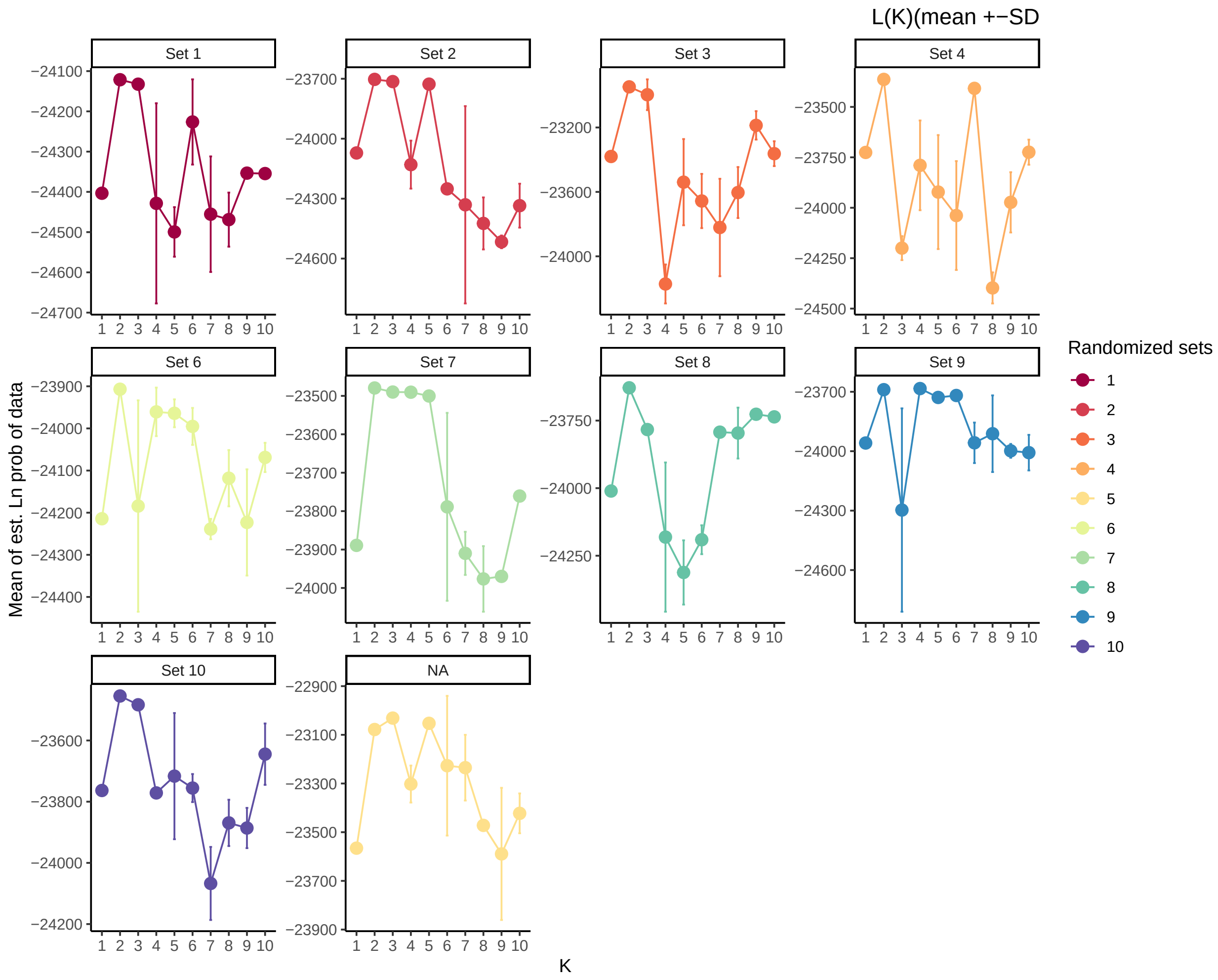

Delta K - Evanno method

1. The mean difference between successive likelihood values of K”:

$$L'(K) = L(K) - L(K-1)$$

2. The (absolute value of the) difference between successive values of L’(K):

$$L''(K)| = |L'(K+1) - L'(K)|$$

3. DetltaK:

$$\Delta K = m(|L(K+1) - 2L(K) + L(K-1)|)/s[L(K)]$$

| a. average the L(K) over the x replicates
| b. estimate from these averages

$$L''(K) = abs(L(K+1) - 2L(K) + L(K-1))$$

c. divide by the standard deviation of L(K) (sd of the different replicates for the same K)

Evanno, G., Regnaut, S., & Goudet, J. (2005). Detecting the number of clusters of individuals using the software STRUCTURE: a simulation study. Molecular ecology, 14(8), 2611-2620.

```
deltaK_sets <- sd_ln_sd %>%
  filter_all(all_vars(set != "ALL SNPs")) %>%
  as_tibble() %>%
  group_by(set) %>%
  mutate("K" = ln.mean, "K-1" = lag(ln.mean, default = 0), "K+1" = lead(ln.mean)) %>%
  mutate("l'(K)" = ln.mean - `K-1`) %>%
  mutate("l''(K)" = abs(`K+1` - 2 * K + `K-1`)) %>%
  mutate(DeltaK = abs((`l''(K)`/ln_sd)))
```

```
#kable(deltaK_sets,  caption = "DeltaK calculation steps")
```

```
deltaK_sets %>%
  ggplot(aes(x = k, y = (DeltaK), color = factor(set))) +
  geom_point(size = 3) +
  geom_line() +
  scale_color_manual("Randomized sets", values=c("#9e0142", "#d53e4f", "#f46d43",
                                                 "#fdae61", "#fee08b", "#e6f598",
                                                 "#abdda4", "#66c2a5", "#3288bd",
                                                 "#5e4fa2"),
                     breaks =c(1,2,3,4,5,6,7,8,9,10)) +
  theme_classic() +
  theme(plot.title = element_text(hjust = 1)) +
  xlab("K") + ylab("Delta K") +
  ggtitle("DeltaK = mean(|L''(K)| / sd(L(K))") +
  xlim("1","2","3","4", "5", "6", "7", "8", "9")
```

```
## Warning: Removed 10 rows containing missing values (geom_point).
```

```
## Warning: Removed 10 row(s) containing missing values (geom_path).
```

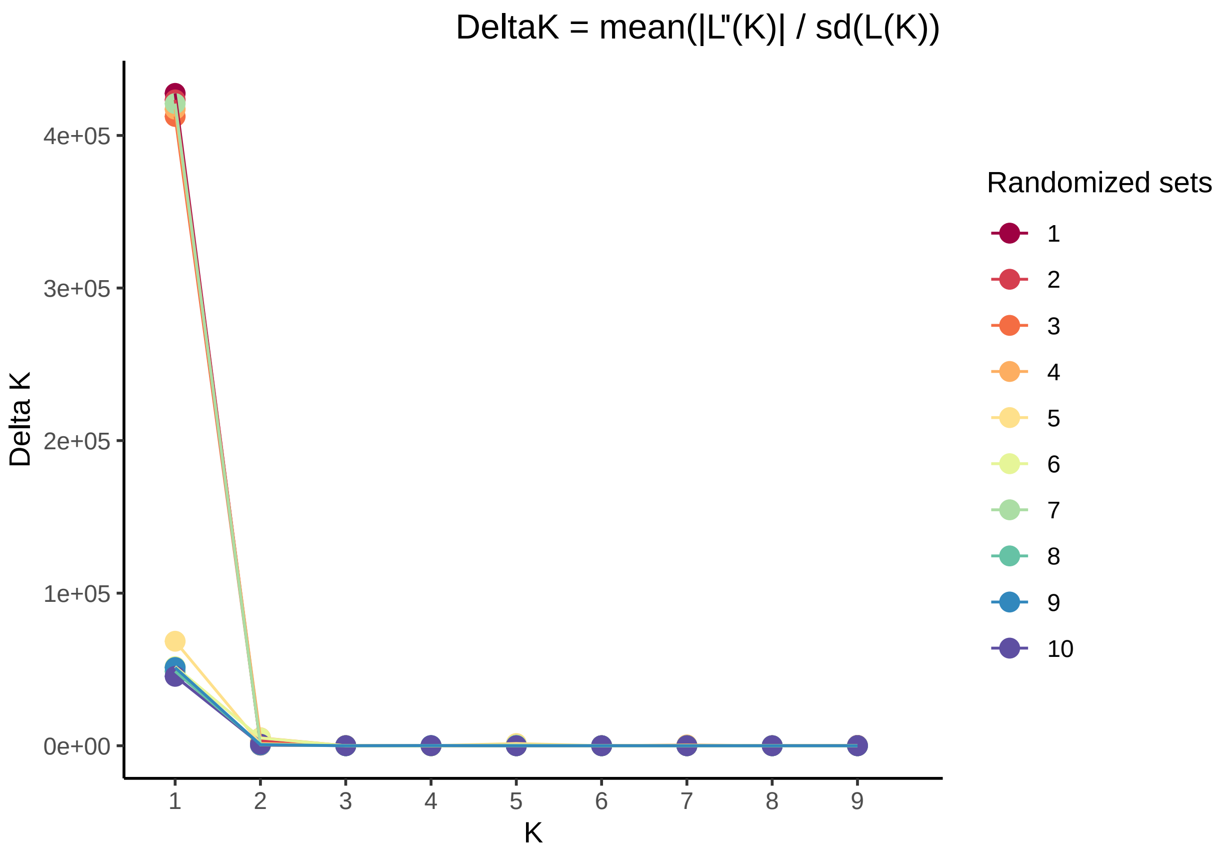

Randomized sets averaged

Datat prep

```
all_sets <- csv %>%
  filter_all(all_vars(set != "ALL SNPs")) %>%
  select(k, Est..Ln.prob..of.data) %>%
  group_by(k) %>%
  mutate(sd = sd(.data$Est..Ln.prob..of.data)) %>%
  summarize(average_ln = mean(Est..Ln.prob..of.data), sd=sd) %>%
  unique()
```

```
## `summarise()` has grouped output by 'k'. You can override using the `.groups`
## argument.
```

```
all_sets %>%
  ggplot(aes(x = k, y = (average_ln))) +
  geom_point(size = 3) +
  geom_line() +
  theme_classic() +
  theme(plot.title = element_text(hjust = 1)) +
  xlab("K") + ylab("Mean of est. Ln prob of data") +
  geom_errorbar(aes(ymin = average_ln - sd, ymax = average_ln + sd), position = "dodge", width = 0.25)
  ggtitle("L(K)(mean +-SD") +
  xlim("1","2","3","4", "5", "6", "7", "8", "9", "10")
```

Table 1: Likelihood mean of the triplicates for each K value. Bars represent standard deviation

k	average_ln	sd
1	-23898.22	292.7619
2	-23537.32	342.7282
3	-23731.25	486.1268
4	-23890.91	358.0051
5	-23796.23	415.5109
6	-23884.83	336.3045
7	-23921.66	407.5969
8	-24003.80	345.1620
9	-23942.28	382.1034
10	-23841.70	336.2670

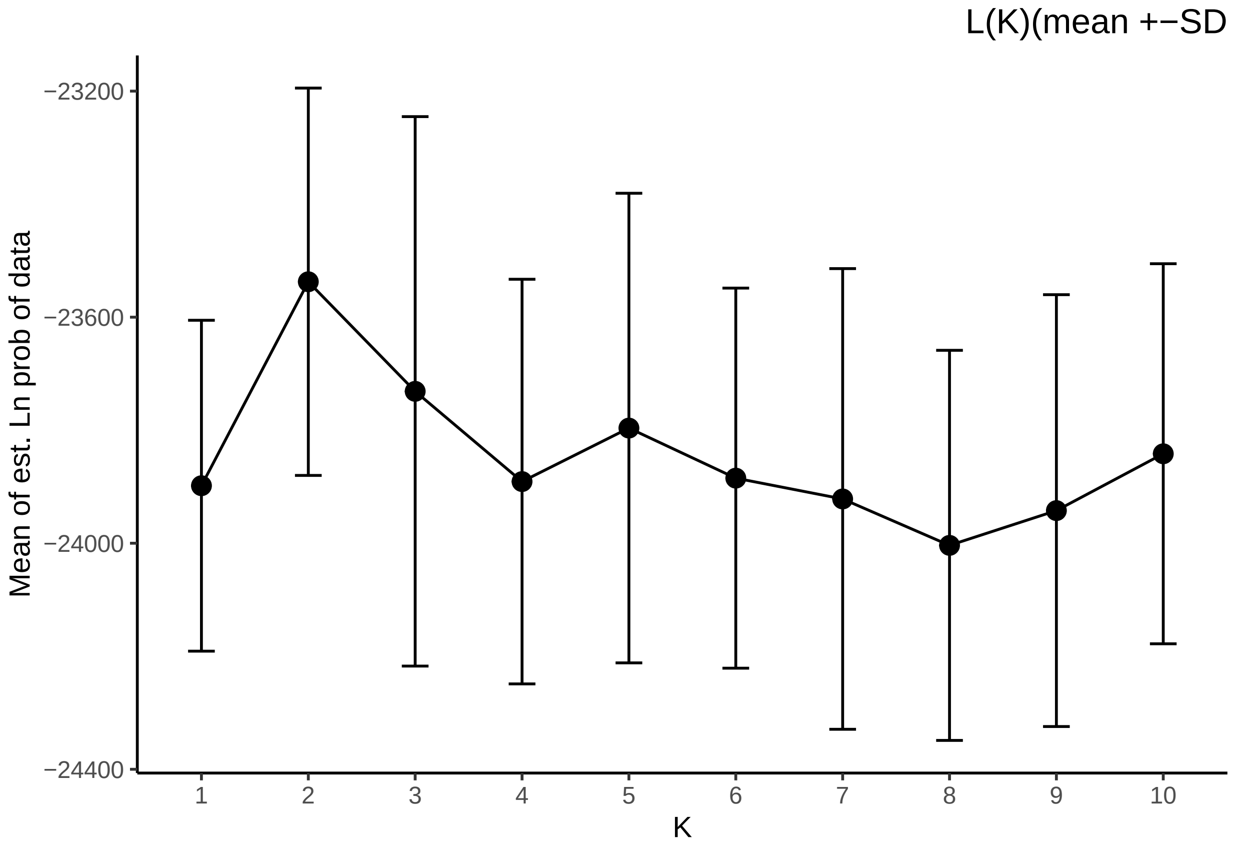


```
kable(all_sets,  caption = "Likelihood mean of the triplicates for each K value. Bars represent standar
```

Delta K - Evanno method

1. The mean difference between successive likelihood values of K":

$$L'(K) = L(K) - L(K-1)$$

2. The (absolute value of the) difference between successive values of L'(K):

Table 2: DeltaK calculation steps

k	average_ln	sd	K	K-1	K+1	l'(K)	l"(K)	DeltaK
1	-23898.22	292.7619	-23898.22	0.00	-23537.32	-23898.22333	24259.13000	82.8629916
2	-23537.32	342.7282	-23537.32	-23898.22	-23731.25	360.90667	554.84333	1.6189018
3	-23731.25	486.1268	-23731.25	-23537.32	-23890.91	-193.93667	34.27667	0.0705097
4	-23890.91	358.0051	-23890.91	-23731.25	-23796.23	-159.66000	254.34000	0.7104367
5	-23796.23	415.5109	-23796.23	-23890.91	-23884.83	94.68000	183.27667	0.4410875
6	-23884.83	336.3045	-23884.83	-23796.23	-23921.66	-88.59667	51.76333	0.1539181
7	-23921.66	407.5969	-23921.66	-23884.83	-24003.80	-36.83333	45.30000	0.1111392
8	-24003.80	345.1620	-24003.80	-23921.66	-23942.28	-82.13333	143.65000	0.4161814
9	-23942.28	382.1034	-23942.28	-24003.80	-23841.70	61.51667	39.06333	0.1022324
10	-23841.70	336.2670	-23841.70	-23942.28	NA	100.58000	NA	NA

$$L''(K)| = |L'(K+1) - L'(K)|$$

3. DetltaK:

$$\Delta K = m(|L(K+1) - 2L(K) + L(K-1)|)/s[L(K)]$$

| a. average the L(K) over the x replicates
| b. estimate from these averages

$$L''(K) = abs(L(K+1) - 2L(K) + L(K-1))$$

c. divide by the standard deviation of L(K) (sd of the different replicates for the same K)

Evanno, G., Regnaut, S., & Goudet, J. (2005). Detecting the number of clusters of individuals using the software STRUCTURE: a simulation study. Molecular ecology, 14(8), 2611-2620.

```
deltaK <- all_sets  %>%
  as_tibble() %>%
  mutate("K" = average_ln, "K-1" = lag(average_ln, default = 0), "K+1" = lead(average_ln)) %>%
  mutate("l'(K)" = average_ln - `K-1`) %>%
  mutate("l''(K)" = abs(`K+1` - 2 * K + `K-1`)) %>%
  mutate(DeltaK = abs((`l''(K)`/sd)))
```

```
kable(deltaK,  caption = "DeltaK calculation steps")
```

```
deltaK %>% ggplot(aes(x = k, y = (`l''(K)`))) +
  geom_point(size = 3) +
  geom_line() +
  theme_classic() +
  theme(plot.title = element_text(hjust = 1)) +
  xlab("K") + ylab("Delta K")+
  ggtitle("DeltaK = mean(|L''(K)| / sd(L(K))") +
  xlim("1","2","3","4", "5", "6", "7", "8", "9")
```

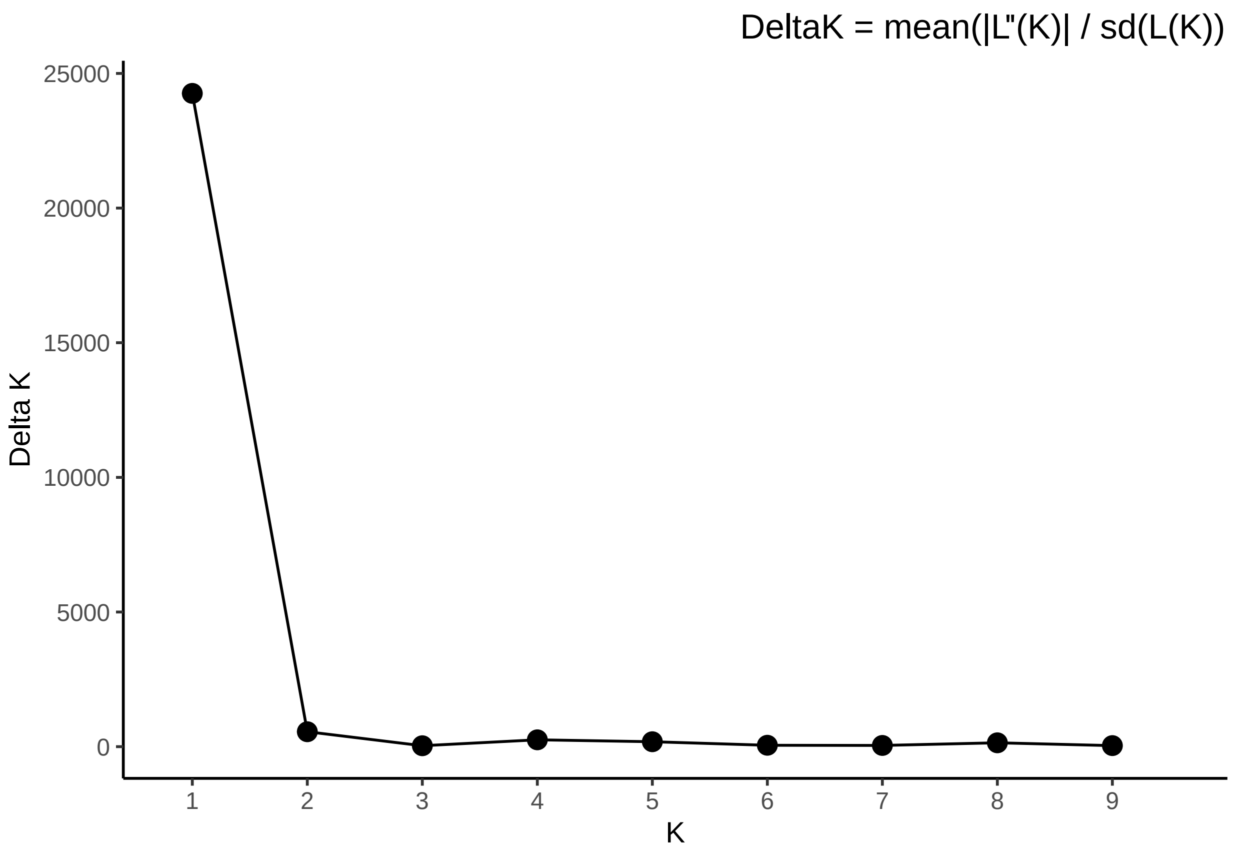

Comparing the admixture values between the full and the randomized data sets

Population level

Overview of the data

```
STD %>%
  select(Population, A1_mean, A2_mean,SD) %>%
  kable(caption = "Averaged Admixture proportions of the 19 populations among the 10 sub-sampled sets ar
```

There is some variation observed in the proportion of membership among the 10 random sets.

```
df_plot <- STD %>%
  right_join(all_snps, by="POP") %>%
  select(Population, A1_mean, A2_mean,A1,A2) %>%
  gather("Set", "Admixed_values", A1_mean:A2) %>%
  mutate(Set = recode(Set, "A1_mean"="Rdm.1","A2_mean"="Rdm.2", "A1"="Full_set.1", "A2"="Full_set.2" ))
  mutate(facet = recode(Set, "Rdm.1"="Rdm","Rdm.2"="Rdm", "Full_set.1"="Full", "Full_set.2"="Full" ))
```

```
hist_plot <- df_plot %>%
  arrange(Population) %>%
  ggplot(aes(fill=Set, y=Admixed_values, x=facet)) +
  geom_bar(position="stack", stat="identity") +
  scale_fill_manual(values = c("#d73027", "#4575b4","#d73027", "#4575b4")) +
  ggtitle("Average add mixted values in the 19 populations") +
```

Table 3: Averaged Admixture proportions of the 19 populations among the 10 sub-sampled sets and the Standard deviation.

Population	A1_mean	A2_mean	SD
TakLan	0.59	0.41	0.05
Xop	0.53	0.47	0.02
MuongHo	0.88	0.12	0.02
MangRuon	0.97	0.03	0.02
TLCenter	0.97	0.03	0.02
MoLut1	0.95	0.05	0.05
TakNgo	0.75	0.25	0.12
ChungTam	0.60	0.40	0.04
TakRang	0.55	0.45	0.03
ConPin	0.70	0.30	0.06
MangLun	0.65	0.35	0.07
TakTui	0.77	0.23	0.06
DacVien	0.80	0.20	0.06
PhuocLo	0.58	0.42	0.05
ChOm	0.92	0.08	0.05
TraCang	0.66	0.34	0.08
TraNam	0.73	0.27	0.09
TraLinh3	0.55	0.45	0.06
LocBong	0.77	0.23	0.04

```
  xlab("") +
  facet_grid(~ Population) +
  facet_wrap(~ Population, nrow=2)

hist_plot +
  theme(panel.spacing = unit(0.5, "lines"))
```

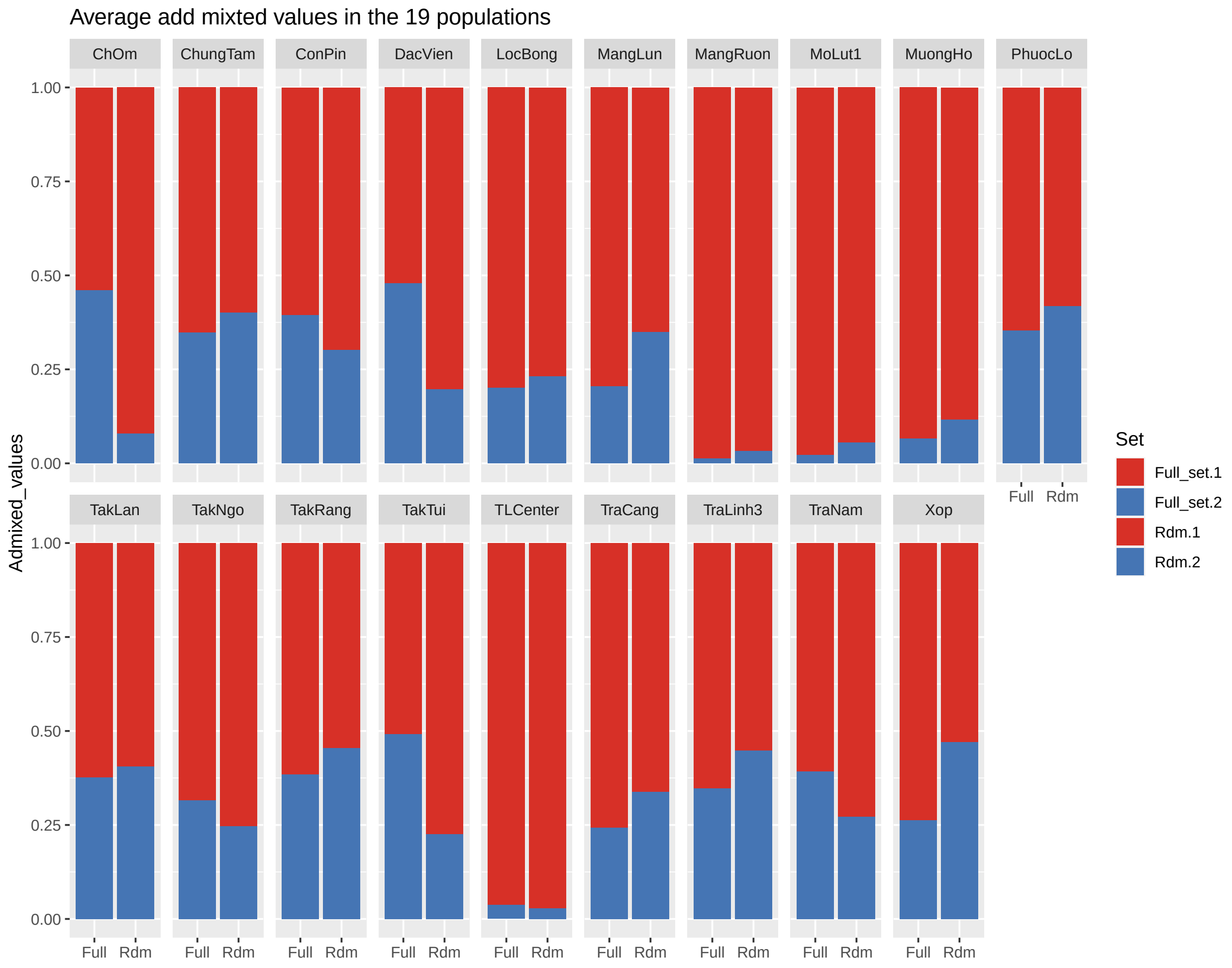

Paired t-test and Wilcoxon signed rank test

```
df_stat <- STD %>%
  right_join(all_snps, by="POP") %>%
  select(Population, A1_mean, A2_mean,A1,A2)
colnames(df_stat)  <-   c("Population", "Rdm.1", "Rdm.2", "Full_set.1", "Full_set.2")

#Preliminary test to check paired t-test assumptions
# Shapiro-Wilk normality test for the differences
d <- df_stat %>% select(Population,Rdm.1, Full_set.1 )

d <- df_stat %>%
  mutate("diff" =  Full_set.1 - Rdm.1)
d <- d$diff
shapiro.test(d) # => p-value = 0.3032
```

```
##
##  Shapiro-Wilk normality test
##
## data:  d
## W = 0.88937, p-value = 0.03137
```

The Shapiro-Wilk p-value (p-value = 0.03137) is lower than the significance level 0.05 implying that the distribution of the differences (d) are significantly different from normal distribution. In other words, we can

not assume normality and we will perform a non-parametric test.

Paired t-test

```
res <- t.test(df_stat$Rdm.1, df_stat$Full_set.1, paired = TRUE)
res
```

```
##
##  Paired t-test
##
## data:  df_stat$Rdm.1 and df_stat$Full_set.1
## t = 0.48707, df = 18, p-value = 0.6321
## alternative hypothesis: true difference in means is not equal to 0
## 95 percent confidence interval:
##  -0.05643259  0.09049574
## sample estimates:
## mean of the differences
##              0.01703158
```

The p-value of the paired t-test is 0.6321, thus greater than the significance level alpha = 0.05. We can then accept the null hypothesis and conclude that the admixture values of the randomized SNPs is not significantly different from the full data set.

Wilcoxon signed rank test

```
wilcox.test(df_stat$Rdm.1, df_stat$Full_set.1, paired = TRUE)
```

```
##
##  Wilcoxon signed rank exact test
##
## data:  df_stat$Rdm.1 and df_stat$Full_set.1
## V = 89, p-value = 0.8288
## alternative hypothesis: true location shift is not equal to 0
```

The p-value of the paired t-test is 0.8288, thus greater than the significance level alpha = 0.05. We can then accept the null hypothesis and conclude that the admixture values of the randomized SNPs is not significantly different from the full data set.

Individual level

Overview of the data

```
df_plotindv <- STDindv %>%
#  right_join(all_snps, by="POP") %>%
  select(Population, indvidual, NA., NA..1,NA..2,NA..3,NA..4,NA..5,NA..6,NA..7,
         NA..8,NA..9,NA..10,NA..11,NA..12,
         NA..13,NA..14,NA..15,NA..16,NA..17,NA..18,NA..19, NA..20, NA..21) %>%
  gather("Set", "Admixed_values", NA.:NA..21) %>%
  mutate(facet = recode(Set, "NA." = "1", "NA..1" = "1", "NA..2" = "2", "NA..3" = "2",
                        "NA..4" = "3", "NA..5" = "3", "NA..6" = "4", "NA..7" = "4",
                        "NA..8" = "5", "NA..9" = "5", "NA..10" = "6", "NA..11" = "6",
                        "NA..12" = "7", "NA..13" = "7", "NA..14" = "8", "NA..15" = "8",
                        "NA..16" = "9", "NA..17" = "9", "NA..18" = "10", "NA..19" = "10",
                        "NA..20" = "full", "NA..21" = "full")) %>%
mutate(Set = recode(Set, "NA." = "Set 1.1", "NA..1" = "Set 1.2", "NA..2" = "Set 2.1",
```

```
                    "NA..3" = "Set 2.2", "NA..4" = "Set 3.2", "NA..5" = "Set 3.1",
                    "NA..6" = "Set 4.1", "NA..7" = "Set 4.2", "NA..8" = "Set 5.1",
                    "NA..9" = "Set 5.2", "NA..10" = "Set 6.1", "NA..11" = "Set 6.2",
                    "NA..12" = "Set 7.2", "NA..13" = "Set 7.1", "NA..14" = "Set 8.1",
                    "NA..15" = "Set 8.2", "NA..16" = "Set 9.1", "NA..17" = "Set 9.2",
                    "NA..18" = "Set 10.1", "NA..19" = "Set 10.2", "NA..20" = "full.1",
                    "NA..21" = "full.2" ))
```

```
hist_plot <- df_plotindv %>%
  arrange(indvidual) %>%
  ggplot(aes(fill=Set, y=Admixed_values, x=facet)) +
  geom_bar(position="stack", stat="identity") +
  scale_fill_manual(values = c("#d73027", "#4575b4","#d73027", "#4575b4",
                               "#d73027", "#4575b4","#d73027", "#4575b4",
                               "#d73027", "#4575b4","#d73027", "#4575b4",
                               "#d73027", "#4575b4","#d73027", "#4575b4",
                               "#d73027", "#4575b4","#d73027", "#4575b4",
                               "#d73027", "#4575b4","#d73027", "#4575b4")) +
  ggtitle("Average add mixted values in the 19 populations") +
  xlab("") +
  facet_grid(~ indvidual) +
  facet_wrap(~ indvidual, nrow=20)

hist_plot +
  theme(panel.spacing = unit(0.5, "lines"))
```

Average add mixted values in the 19 populations

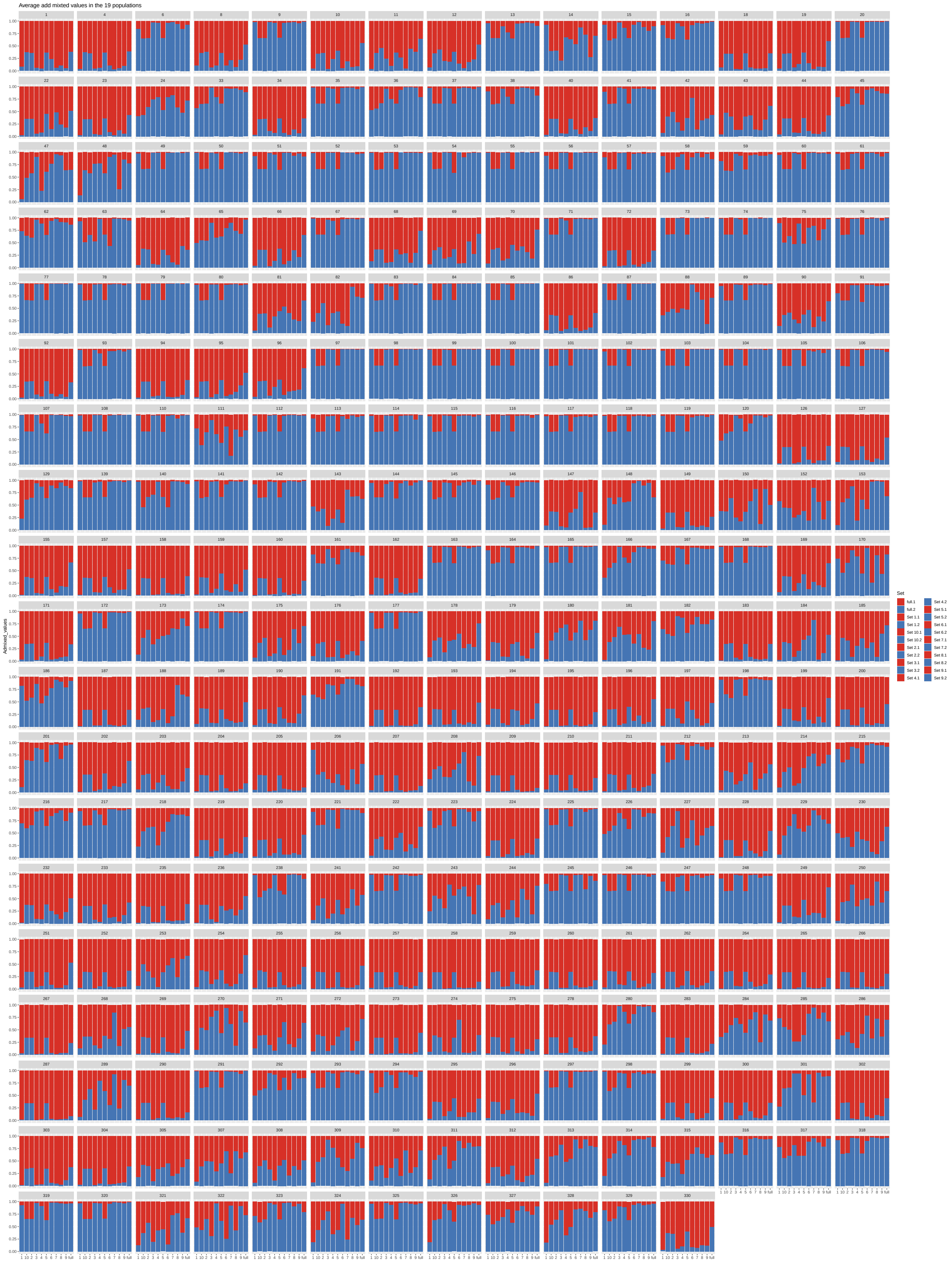

Pairwise t-test

```
df_stat <- df_plotindv %>%
  select(indvidual, Set, Admixed_values) %>%
  filter( Set != "Set 1.2") %>%
  filter( Set != "Set 2.2") %>%
  filter( Set != "Set 3.2") %>%
  filter( Set != "Set 4.2") %>%
  filter( Set != "Set 5.2") %>%
  filter( Set != "Set 6.2") %>%
  filter( Set != "Set 7.2") %>%
  filter( Set != "Set 8.2") %>%
  filter( Set != "Set 9.2") %>%
  filter( Set != "Set 10.2") %>%
  filter( Set != "full.2")
pwc <- df_stat %>% pairwise_t_test( Admixed_values ~ Set, paired = TRUE,
                                   p.adjust.method = "bonferroni")
pwc <- pwc %>%  filter( group1 == "full.1")
pwc  %>%
  select(group1,group2, n1, n2, statistic,  df, p,  p.adj,  p.adj.signif)%>%
  kable(format="latex", digits = 32)
```

group1	group2	n1	n2	statistic	df	p	p.adj	p.adj.signif
full.1	Set 1.1	282	282	-18.55445	281	0.00e+00	0.00e+00	****
full.1	Set 10.1	282	282	-24.14482	281	0.00e+00	0.00e+00	****
full.1	Set 2.1	282	282	-22.35093	281	0.00e+00	0.00e+00	****
full.1	Set 3.1	282	282	-13.34317	281	9.00e-32	4.72e-30	****
full.1	Set 4.1	282	282	-16.33693	281	0.00e+00	0.00e+00	****
full.1	Set 5.1	282	282	-23.27661	281	0.00e+00	0.00e+00	****
full.1	Set 6.1	282	282	-11.59198	281	1.18e-25	6.49e-24	****
full.1	Set 7.1	282	282	-10.07171	281	1.43e-20	7.86e-19	****
full.1	Set 8.1	282	282	-12.56183	281	5.03e-29	2.77e-27	****
full.1	Set 9.1	282	282	-13.16375	281	3.70e-31	2.06e-29	****

Wilcoxon signed rank test

```
pwt <- df_stat %>%
  wilcox_test( Admixed_values ~ Set, paired = TRUE,
              p.adjust.method = "bonferroni") %>%
  filter( group1 == "full.1")

pwt  %>%
  select(group1,group2, n1, n2, statistic, p,   p.adj,  p.adj.signif)%>%
  kable(format="latex", digits = 32)
```

group1	group2	n1	n2	statistic	p	p.adj	p.adj.signif
full.1	Set 1.1	282	282	1466.0	0.00e+00	0.00e+00	****
full.1	Set 10.1	282	282	1467.5	0.00e+00	0.00e+00	****
full.1	Set 2.1	282	282	1755.5	0.00e+00	0.00e+00	****
full.1	Set 3.1	282	282	6073.0	1.17e-23	6.43e-22	****
full.1	Set 4.1	282	282	2910.5	0.00e+00	0.00e+00	****
full.1	Set 5.1	282	282	1595.0	0.00e+00	0.00e+00	****
full.1	Set 6.1	282	282	6857.0	1.26e-21	6.93e-20	****
full.1	Set 7.1	282	282	8969.5	4.93e-15	2.71e-13	****
full.1	Set 8.1	282	282	6103.5	8.90e-24	4.89e-22	****
full.1	Set 9.1	282	282	4998.5	1.05e-27	5.78e-26	****

Results and dicussion

Among the STRUCTURE triplicates, we observe a high standard deviation on the likelihood estimates. The sub-sampling has reduced the number of SNPs and this introduces some stochasticity in the analysis. Despite these variations, all the sets converged to an optimal number of population of two. We compiled the average admixture value for each population. The 10 different sets provided us the same admixted values, without exception. The results of the sub-sampling sets are consistent. The comparison of the averaged admixture proportions in the 19 populations between the sub-sampled and the full data sets, shows that the admixture values of the randomized SNPs are not significantly different from the full data set (p-value = 0.6321). The comparison of the averaged admixture proportions at the individual level between the sub-sampled and the full data sets, shows that the admixture values of the randomized SNPs are significantly different from the full data set.

The potential presence of linked markers in our full data set does not affect the STRUCTURE estimation of number of ancestral populations. However, as we are using a small set of SNPs, we observe more stochasticity at the individual level, where the estimate of the admixture values are not always consistent and statistically not comparable. It is difficult to estimate if that result is the consequence of the reduction of number of SNPs or the effect of linked SNPs.
